# Impact of physical and sexual abuse on risk of hospitalisations for physical and mental illnesses: insights from two large prospective cohort studies

**DOI:** 10.1016/j.lanepe.2024.100883

**Published:** 2024-03-12

**Authors:** Philipp Frank, G. David Batty, Jaana Pentti, Markus Jokela, Jenni Ervasti, Andrew Steptoe, Glyn Lewis, Mika Kivimäki

**Affiliations:** a UCL Brain Sciences, University College London, 149 Tottenham Court Rd, London, W1T 7BN, UK; b Research Department of Epidemiology and Public Health, University College London, 1-19 Torrington Place, London, WC1E 6BT, UK; c Clinicum, Faculty of Medicine, University of Helsinki, Tukholmankatu 8 B, Helsinki, FI-00014, Finland; d Department of Public Health, University of Turku, Turku, Finland; e Finnish Institute of Occupational Health, Topeliuksenkatu 41 B, Helsinki, FI-00250, Finland; f Department of Psychology and Logopedics, Faculty of Medicine, University of Helsinki, Haartmaninkatu 3, Helsinki, 00290, Finland; g Research Department of Behavioural Science and Health, University College, London, 1-19 Torrington Place, London, WC1E 7HB, UK

**Keywords:** Physical abuse, Sexual abuse, Mental disorders, Physical illness, Hospitalisations, Cohort study

## Abstract

**Background:**

Physical abuse can lead to severe health consequences that extend beyond immediate harm. We explored the associations of physical abuse experienced during childhood and adulthood with a wide range of adult health conditions requiring hospital treatment.

**Methods:**

We utilised data from a sub-cohort of 157,366 UK Biobank participants (46.4% of the baseline population; age range 45–81; 89,101 women) and repeated analyses in an independent population of 85,929 adults from the Finnish Public Sector (FPS) study (age range 17–78; 68,544 women). Participants in both cohorts reported instances of physical and sexual abuse at study baseline. Follow-up included 77 common health conditions ascertained from linkage data to national hospital and mortality registries.

**Findings:**

Mean follow-up duration was 4.6 years (SD 0.14) in UK Biobank and 10.6 years (4.3) in FPS. Physical and sexual abuse was associated with 22 mental and physical health conditions. After multivariable adjustments, participants who experienced abuse during both early and later stages of life had a 2.12- (95% confidence interval 1.39–3.23) to 3.37-fold (1.52–7.45) increased risk of mental and behavioural disorders, a 1.46 (1.20–1.79) to 1.83 (1.05–3.20) times increased risk of metabolic, haematologic, and respiratory diseases, and a 1.24 (1.07–1.45) times higher risk of inflammatory diseases compared with non-exposed participants. The absolute risk difference between these groups was greatest for metabolic and haematologic conditions (rate 381 and risk difference 160 per 100,000 person-years). Frailty, comorbidities, and competing risk of death did not modify these associations, but the possibility of bias or residual confounding cannot be excluded.

**Interpretation:**

Repeated exposure to physical and sexual abuse amplifies the risk of hospitalisations from mental disorders and physical diseases spanning diverse organ systems. Addressing this issue may necessitate multifaceted strategies, including shifts in societal norms, legal measures, and increased healthcare provision for affected individuals and their families.

**Funding:**

Wellcome Trust, UK Medical Research Council, 10.13039/100000049U.S. National Institute on Aging, 10.13039/501100002341Academy of Finland.

**Research in context**

**Evidence before this study**

We searched PubMed for studies exploring the associations of physical abuse with mental and physical health outcomes, without language or date restrictions, from database inception until February 5, 2024, using the search terms “physical abuse”, “sexual abuse”, “intimate partner violence”, “adverse childhood experiences”, “health”, “morbidity”, and “mortality”. Of the 8535 identified articles, we found no large-scale studies that explored the associations between physical abuse exposure across the life course and a broad range of health outcomes within a single analytical setting. Such outcome-wide studies would facilitate non-selective reporting, a systematic assessment of disease specificity, and avoid methodological and statistical heterogeneity observed in previous meta-analytic reviews of multiple disease outcomes.

**Added value of this study**

This study investigated the risk of hospitalisation for 77 common health conditions in individuals who had experienced physical abuse during childhood, adulthood, or at both stages of life. Using longitudinal data from up to 156,958 participants of the UK Biobank study and 85,929 participants from the Finnish Public Sector study, we found that exposure to physical abuse in childhood or adulthood was associated with an increased risk of 22 health conditions requiring hospital treatment, including mental and behavioural disorders; diseases of the metabolic, haematologic, musculoskeletal, circulatory, and respiratory system; as well as infections. These associations were particularly strong in individuals who had experienced physical abuse during both childhood and adulthood. Regardless of the timing of abuse, relative risks were highest for hospitalisations due to mental and behavioural disorders. The greatest absolute differences between individuals with and without a history of abuse were observed for metabolic and haematologic conditions. Most of these associations were partially attributable to depressive symptoms, obesity, smoking, and inflammation.

**Implications of all the available evidence**

Preventing physical and sexual abuse is a complex task that requires evidence-based interventions targeting various domains, ranging from societal norms and legislative measures to individual and familial aspects. In health care, routine screening procedures and effective early detection systems are needed to identify victims of abuse and provide trauma-informed services. Underlining the importance of abuse prevention, our findings show that physical and sexual abuse is associated with a wide range of severe mental and physical illnesses requiring hospital treatment, and that repeated exposure to abuse across the life course may further exacerbate the risk of ill health.

## Introduction

Despite substantial efforts aimed at its eradication, physical and sexual abuse continues to be a persistent issue in societies, affecting individuals in both early life and adulthood.^1^, ^2^, ^3^ Globally, it is estimated that one in five children has been exposed to physical abuse, while one in ten has endured sexual abuse.^1^ In the United States, almost one third of adult women report having experienced sexual or physical violence by an intimate partner.^4^

Recent findings from large epidemiological studies suggest long-term associations of childhood and adulthood abuse with mental and behavioural disorders, selected physical illnesses, and premature mortality.^5^, ^6^, ^7^, ^8^, ^9^ This evidence base, however, has not comprehensively examined the full scope of disease categories associated with physical and sexual abuse experienced during childhood, adult life, or, crucially, at both stages of life. Studies in this field have typically focused on a limited set of specific disease endpoints, with separate emphasis on either childhood or adulthood abuse as the sole exposure of interest.^10^, ^11^, ^12^, ^13^, ^14^, ^15^ These studies have utilised different populations, settings, and designs, as well as heterogeneous measures of abuse and health outcomes,^2^^,^^5^^,^^16^^,^^17^ complicating a comparison of the effects of abuse on different disease outcomes. In addition, many studies have included physical and sexual abuse among broader estimates of adverse childhood experiences rather than focusing on the long-term health outcomes associated with each adversity separately.^18^

Using data from the UK Biobank, we conducted a large-scale, outcome-wide study to examine the associations of childhood and adulthood physical and sexual abuse with the incidence of 77 common health conditions requiring hospital treatment; test the extent to which these associations are attributable to poorer risk profiles of individuals with a history of physical or sexual abuse; and explore the reproducibility of findings by repeating the main analyses in an independent dataset of Finnish adults.

## Methods

### Study populations

In our primary analysis, we utilised data from the UK Biobank study, a large prospective cohort study.^19^^,^^20^ Among the 9.1 million adults eligible for inclusion in UK Biobank, a total of 502,665 aged 38–73 years (273,450 women, 229,215 men) participated in a baseline clinical examination between 2006 and 2010. Of these, 339,092 were re-invited via email to fill out a series of online follow-up questionnaires. A subgroup of 157,366 participants (response rate: 46.4%) completed an online follow-up mental health questionnaire when information on physical and sexual abuse was collected for the first time. Taking place in 2016–17, this represents the study baseline for the present analysis.

To test the reproducibility of our findings from UK Biobank, we repeated our main analyses in an independent cohort, the Finnish Public Sector Study (FPS). FPS is an occupational cohort study consisting of 113,578 Finnish employees aged 17–78 years who had completed a survey during 2000 to 2002, 2004 to 2005, 2008 to 2009, and/or 2011 to 2013. Of these, we included 85,929 individuals (68,544 women, 17,385 men) who had no missing data on the exposures and covariates of interest and were successfully linked to records of national health registries.

This study followed the Strengthening the Reporting of Observational Studies in Epidemiology (STROBE) reporting guideline for cohort studies. Data collection in UK Biobank was approved by the NHS National Research Ethics Service, and in FPS, by the ethics committee of the Finnish Institute of Occupational Health. In both cohorts, participants provided written consent before their participation in data collection.

### Sexual and physical abuse

Sexual and physical abuse during childhood and adulthood were assessed at study baseline. In the primary analysis of UK Biobank participants, data on childhood physical and sexual abuse were ascertained from two questions taken from the brief Childhood Trauma Questionnaire.^21^ These enquiries ascertained if individuals had experienced severe physical abuse causing bruises or marks by a family member (“People in my family hit me so hard that it left me with bruises or marks”) and/or childhood sexual molestation (“Someone molested me sexually”). Adulthood partner abuse was evaluated using two questions adapted from the British Crime Survey,^22^ in which participants reported their experience of physical violence or other forms of violence (“A partner or ex-partner deliberately hit me or used violence in any other way”), and/or sexual interference or forced sexual activity by a partner or ex-partner (“A partner or ex-partner sexually interfered with me, or forced me to have sex against my wishes”). Both childhood and adulthood inventories used the same response scale (range: 0 for never to 4 for very often). In FPS, data on physical or sexual violence was drawn from a single question regarding participants exposure to physical or sexual abuse (0 for not at all, 1 for during the past 12 months, 2 for more than 12 months ago).

In both UK Biobank and FPS, we computed separate binary variables (yes/no) for each abuse item such that a score of 1 or higher indicated that the person had experienced abuse, while a score of zero represented the reference group of individuals who had not been exposed to such adversities.

Although the primary focus of this study is on physical and sexual abuse experienced across the life course, additional analyses exploring the associations between emotional and physical neglect—two common adverse childhood experiences—and an array of physical and mental health endpoints are available in the Supplement (pp 30–31).

### Covariates

We used an array of covariates captured at baseline. Our selection of covariates was informed by previous research in this field.^5^^,^^8^^,^^23^ Sociodemographic variables were age and sex. Self-reported ethnic/racial origin was included as an additional covariate, with participants choosing among the categories Asian/Asian British, Black/Black British, Chinese, White, mixed (multiracial), and other ethnic/racial groups. However, due to a majority of UK Biobank participants having a White ethnic/racial origin, there were insufficient health events within individual minority groups for meaningful analysis. As a result, we categorised the data as either White or non-White. Educational qualification (none/elementary, secondary, tertiary) was used as an indicator of socioeconomic position.

We also measured other potential confounders and mediators of the abuse–disease link.^5^^,^^6^^,^^8^ Health behaviours included self-reported smoking (never vs previous or current smoker), alcohol consumption (up to three or four times per week vs daily or almost daily), and physical inactivity (physically active vs not active). Self-reported depressive symptoms were ascertained from the 9-item version of the Patient Health Questionnaire, with scores of 10 or higher indicating probable depression.^24^ Body mass index (BMI) was computed from weight in kilograms divided by height in meters squared (kg/m^2^). Obesity was denoted as a BMI of ≥30 kg/m^2^. C-reactive protein (CRP, mg/L) was included as a marker of systemic inflammation and, in our analyses, log-transformed owing to its skewed distribution. In mediation analyses of the association between early-life sexual and physical abuse and disease risk, adulthood partner abuse (no vs sexual/physical abuse) was examined as an additional mediating mechanism.

### Follow-up of conditions requiring hospital treatment

Ascertainment of hospital-treated conditions before or at baseline and incident hospital-treated conditions at follow-up was based on linked electronic health records. Participants in UK Biobank and FPS were linked to national hospital (in the UK, NHS Hospital Episode Statistics) and mortality registers (in the UK, NHS Central Registry). These electronic health records provided information on newly developed physical and mental health conditions that required hospital treatment and, where applicable, death by cause. Event surveillance was from January 1996 until December 2018 for a mean (SD) of 10.6 (4.3) years in the Finnish study, and July 2016 until May 2021 for a mean (SD) of 4.6 (0.14) years in UK Biobank.

In both UK Biobank and FPS, individual diseases and disease categories were coded according to the International Classification of Diseases 10th Revision (ICD-10). We examined 77 specific disease chapters and diagnostic groups. For each health outcome, participants with the disease at or before baseline, as ascertained from the linked health registry data, were excluded from the analysis of incident hospital-treated conditions at follow-up.

### Statistical analysis

**Fig. 1:**
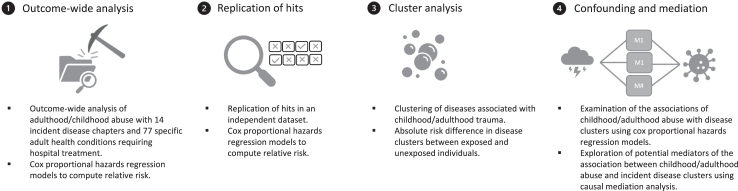
**Design of the study**.

Our analysis, illustrated in Fig. 1, involved the following steps. First, in the primary analyses of UK Biobank participants, after confirmation that the proportional hazards assumptions had not been violated (Supplement, pp 3–13), we computed separate Cox proportional hazards regression models^25^ to examine the associations of each abuse measure with 14 main ICD-10 disease chapters. Hazard ratios (HRs) and accompanying 95% confidence intervals (CIs) were adjusted for age and sex. We restricted subsequent analyses to nine disease chapters that were associated with at least two of the four abuse exposures (*i.e.,* childhood physical abuse, childhood sexual abuse, adulthood physical abuse, and adulthood sexual abuse; p < 0.05).

Next, in a higher resolution analysis, we performed a series of additional survival analyses to investigate the associations of each abuse measure with specific disease endpoints captured within the nine ICD-10 disease chapters. Non-overlapping disease endpoints that showed the most robust links with childhood and adulthood abuse in UK Biobank were selected for subsequent analyses. Robustness (at least 3 of the 5 criteria met) was evaluated based on the magnitude of the effect (“yes”, a HR ≥ 1.20; otherwise “no”); statistical signifcance at P < 0.05 after adjustment for age and sex (“yes,”; otherwise “no”); consistency across exposures (“yes,” association with endpoint evident across ≥2 exposures; otherwise “no”), statistical significance after controlling for multiple testing at a Bonferroni-corrected α level of P < 6.49 × 10−4 (i.e., adjustment for 77 tests; “yes”, the association remains after multiple testing; otherwise “no”); and replication of exposure-outcome associations in an independent population, employing the same statistical adjustments and examining identical disease endpoints (“yes”, replication of association in FPS; otherwise “no”). In addition, to confirm that the observed associations were not sex-specific, we entered an interaction term ‘abuse × sex’ in a model which also included their main effects.

We then scrutinised how disease endpoints that were associated with exposure to sexual and physical abuse in both primary and replication grouped together in individuals with a history of abuse, using phi correlation coefficients and hierarchical cluster analysis.^26^ In a further set of survival analyses, we examined whether the associations of childhood abuse, adulthood abuse, and repeated exposure to both childhood and adulthood abuse with the identified disease clusters remained after multivariable adjustment. Accordingly, we controlled effect estimates for age and sex, and additionally for ethnic/racial origin and educational attainment. To assess the risk differences in disease occurrence between individuals with and without a history of abuse in absolute terms, we calculated the multivariable adjusted rate per 100,000 person-years for each disease cluster.

To evaluate potential bias arising from the low response rate to UK Biobank's online follow-up (46.4%), we performed a series of post-hoc analyses. These analyses examined whether features that may characterise non-respondents, such as frailty, comorbidity, and mortality, are likely to affect the associations between abuse and disease endpoints. First, we conducted separate Cox proportional hazards regression models with interaction terms between lifecourse exposure to physical/sexual abuse and past or present comorbidities, as well as between lifecourse exposure to physical/sexual abuse and frailty. Details on the measurement of comorbidities and frailty^27^ are provided in the Supplement (p 14). Second, we used the Fine and Gray method^28^ (proportional sub-hazards model) to examine the extent to which accounting for the competing risk of death affected the abuse-disease associations.

In addition, we conducted separate causal mediation analyses to examine the mediating role of smoking, alcohol consumption, physical inactivity, obesity, depressive symptoms, and C-reactive protein in the abuse–disease cluster relationships.^29^ Causal mediation analysis dissects the total effect of abuse into two distinct components: the direct effect, which operates independently of any mediator, and the indirect effect, which operates through one or more mediators to influence the outcome.

All UK Biobank analyses were conducted using Stata version 17.0 and RStudio version 2023.03.1 (R package ‘*regmedint’*^29^ for causal mediation analysis), and those of FPS using SAS version 9.4. Statistical code is provided in the Supplement (pp 15–16).

### Role of the funding source

The funders of the study had no role in study design, data collection, data analysis, data interpretation, or writing of the report.

## Results

**Fig. 2:**
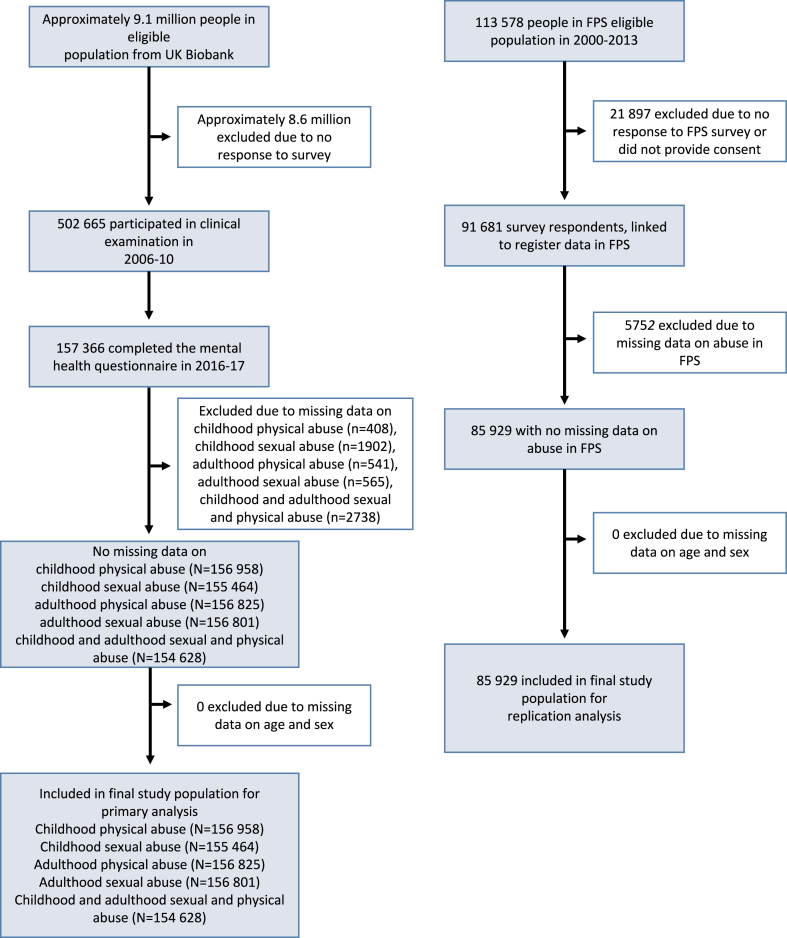
**Study profile**.

Analytical samples in UK Biobank varied according to the exposure of interest (Fig. 2). Of the 157,366 UK Biobank participants who responded to the follow-up questionnaire on mental health (46.4% of those invited), 122,123 (66,437 women, 54.4%) had complete data on the exposures and covariates and were successfully linked to electronic records of national health registries. The mean (SD) age of this sample was 63.3 (7.6) years. Out of these, 30,157 (24.7%, 54.4% women) reported childhood sexual or physical abuse, 18,318 (15.0%, 74.3% women) experienced adult violence from a partner or ex-partner, and 7632 (8.6%, 72.8% women) reported having experienced both. Compared with unexposed individuals, those with a history of abuse were more likely to be women, younger in age, and current smokers at baseline. They were also more likely to have an ethnic/racial minority background, higher levels of C-reactive protein, and to report depressive symptoms. By contrast, they were less likely to consume alcohol on more than 3 days per week compared with their unexposed counterparts. No differences in physical activity levels between these two groups were observed. Differences in 2006/10 baseline characteristics between the baseline sample and analytical (i.e., follow-up) sample are shown in the Supplement (p 19, and below). While there was no apparent difference in the proportion of men and women between the two samples, participants who completed the online follow-up questionnaire appeared to have a slightly more favourable risk factor profile.

In the replication cohort, the FPS, the analytical sample consisted of 85,929 individuals (68 544 women [79.8%]), with a mean (SD) age of 45.3 (10.7) years. Among these, 14,650 (17.1%; of whom 12,279 were women [83.8%]) had a self-reported history of physical or sexual abuse. Further information on baseline characteristics of the primary and replication cohorts is provided in the Supplement (pp 17–19).

**Fig. 3:**
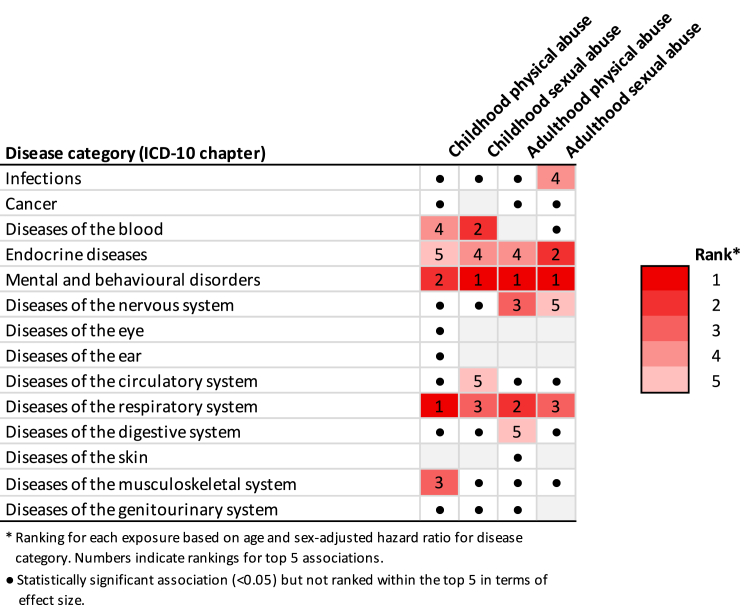
**Association of childhood and adulthood sexual/physical abuse with main ICD-10 disease chapters according to rank (UK Biobank)**.

In Fig. 3, we rank (1–5) the associations of each abuse measure with 14 main ICD-10 disease chapters according to statistical significance and strength in UK Biobank. Exposure to abuse in both early and later life was associated with nine disease chapters. After adjustment for age and sex, the strongest associations of physical and/or sexual abuse in childhood and adulthood were observed for hospitalisations due to mental and behavioural disorders (hazard ratio [HR] range: 1.28–2.40), followed by diseases of the respiratory (range: 1.32–1.37) and endocrine (range: 1.20–1.42) systems. Less consistent associations were found for hospital-treated cancers, diseases of the eye, ear, and skin, as well as diseases of the genitourinary system, which we therefore excluded from subsequent analyses.

**Fig. 4:**
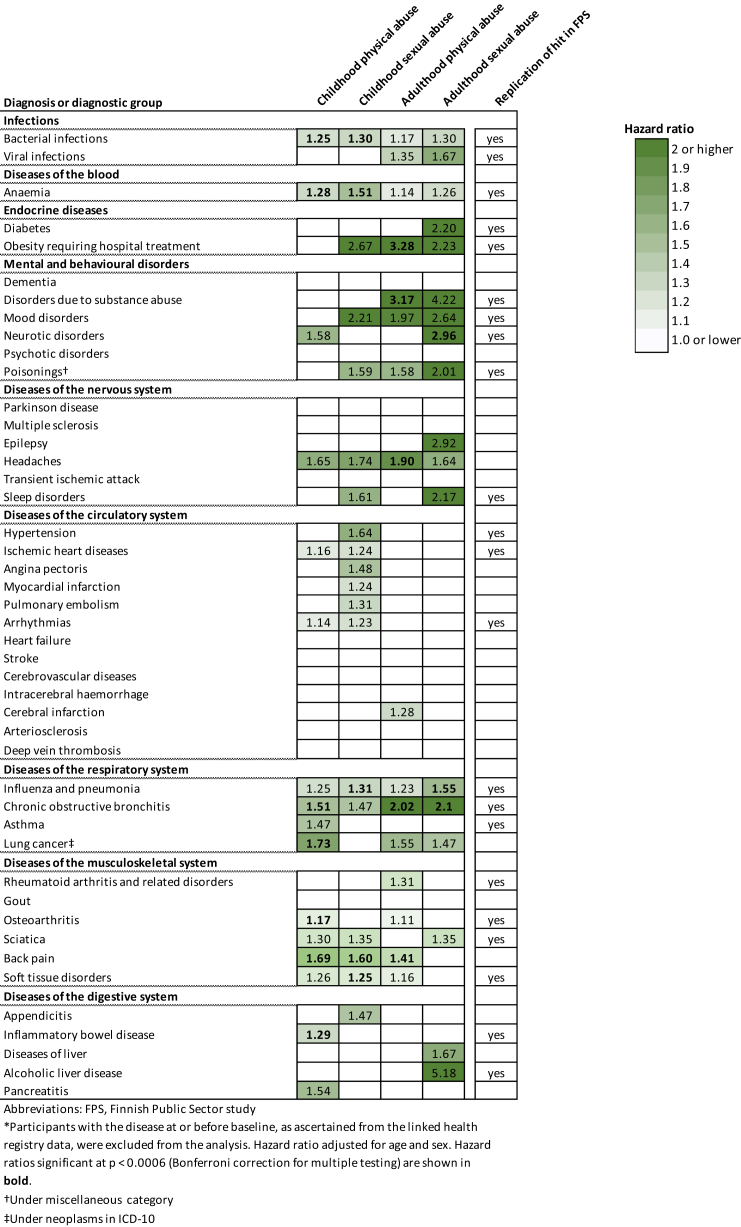
**Association of childhood and adulthood physical and sexual abuse with incident disease (UK Biobank and FPS)**.

In Fig. 4, we present the associations of physical and sexual abuse with specific disease endpoints within the nine selected ICD-10 disease chapters that were associated with abuse in the previous analyses. In UK Biobank, sexual and physical abuse in early and later stages of life was associated with 33 distinct disease endpoints (results for all 77 disease outcomes are reported in the Supplement, pp 20–22). There were no consistent sex differences across abuse indicators, although men showed a stronger association between adulthood sexual abuse and diabetes (HR and 95% CI in women vs men 1.27, 0.54–1.98 vs 7.62, 3.08–18.83). For women, adulthood physical abuse had a stronger link with bacterial infections (1.27, 1.10–1.46 vs 0.97, 0.77–1.12) and chronic obstructive bronchitis (2.51, 1.85–3.40 vs 1.23, 0.74–2.05) compared with men.

Analyses of the Finnish cohort data confirmed 22 of the 33 associations observed in the primary analysis, including bacterial (HR 1.33, 95% CI, 1.19–1.48) and viral (1.36, 1.04–1.78) infections; anaemia (1.40, 1.04–1.97); diabetes (1.21, 1.12–1.31); hospital-treated obesity (1.87, 1.46–2.39); disorders due to substance abuse (2.30, 1.83–2.89); mood (2.37, 1.96–2.86) and neurotic (2.44, 2.08–2.86) disorders; hospital-treated poisonings (2.83, 2.23–3.60); sleep disorders (1.57, 1.42–1.73); hypertension (1.12, 1.03–1.21); ischemic heart disease (1.14, 1.02–1.28); arrythmias (1.10, 1.01–1.21) influenza and pneumonia (1.28, 1.14–1.44); chronic obstructive bronchitis (1.72, 1.36–2.17); asthma (1.37, 1.25–1.51); rheumatoid arthritis and related disorders (1.17, 1.12–1.23); osteoarthritis (1.13, 1.04–1.22); sciatica (1.22, 1.05–1.43); soft tissue disorders (1.19, 1.09–1.29); as well as inflammatory bowel (1.22, 1.05–1.43) and alcoholic liver (1.69, 1.13–2.51) disease (results for all 77 disease outcomes in FPS are reported in the Supplement, pp 23–24). While, in the UK Biobank cohort, most of these associations were evident across all four abuse measures, an increased risk of hospitalisations due to circulatory conditions was primarily observed in individuals with a history of childhood physical or sexual abuse.

**Fig. 5:**
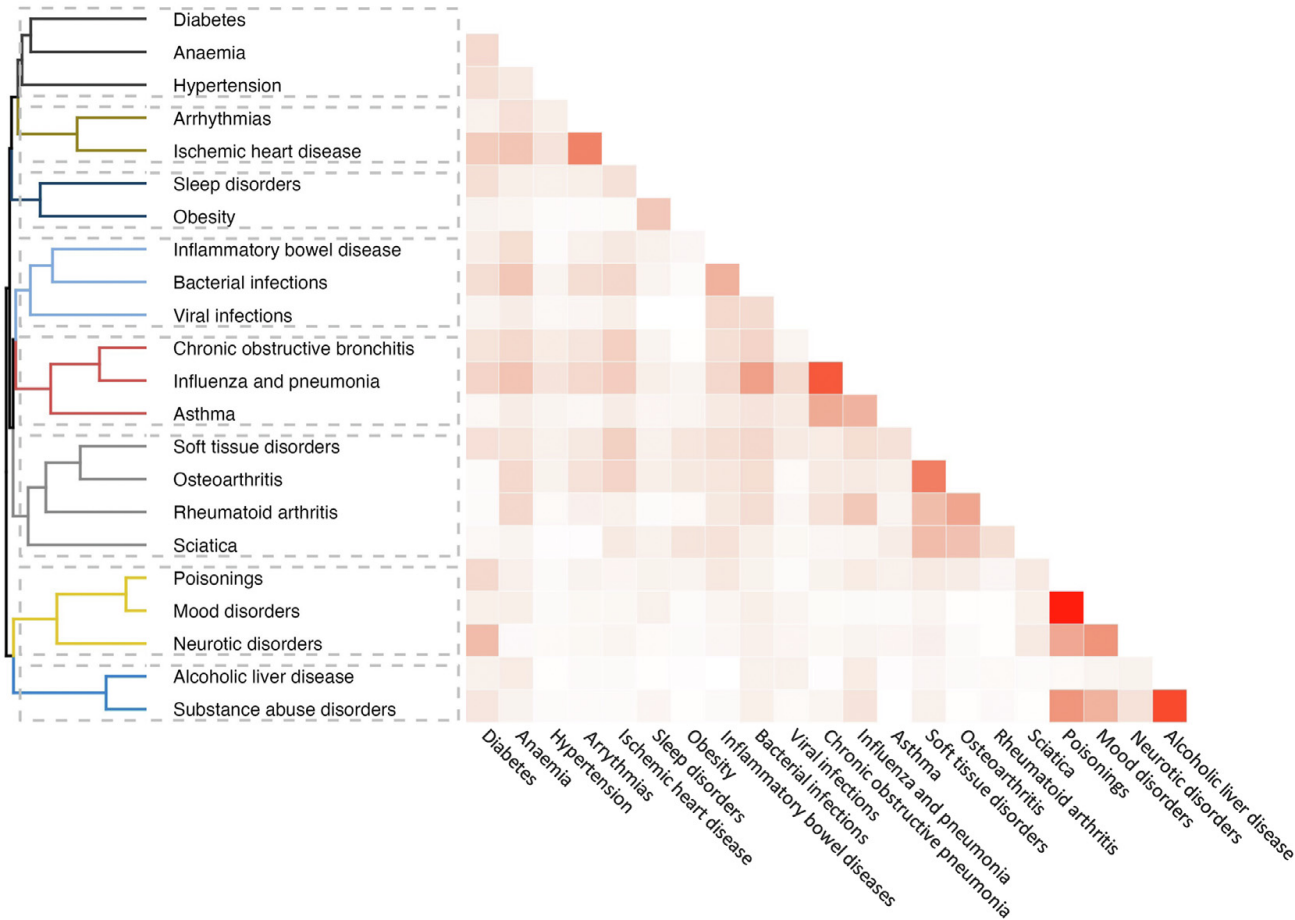
**Patterns of disease clusters associated with physical and sexual abuse during childhood and adulthood (UK Biobank)**.

The clustering of these 22 health conditions in UK Biobank participants who had encountered instances of physical or sexual abuse during childhood and/or adulthood is shown in Fig. 5. We identified 8 disease clusters: cluster 1 encompassed three metabolic and haematologic disorders (diabetes, anaemia, and hypertension); cluster 2 consisted of sleep disorders and obesity; cluster 3 of inflammatory diseases (inflammatory bowel disease, bacterial infections, and viral infections); cluster 4 of respiratory diseases (chronic obstructive bronchitis, influenza, pneumonia, and asthma); cluster 5 of musculoskeletal conditions (soft tissue disorders, osteoarthritis, rheumatoid arthritis, and sciatica); cluster 6 of hospital-treated poisonings, mood disorders, and neurotic disorders; cluster 7 of disorders due to substance abuse and alcoholic liver disease; and cluster 8 of cardiac disorders (ischaemic heart disease and arrhythmias).

Table 1 shows the results of the analyses examining the strength of associations between abuse and 7 disease clusters for individuals who experienced abuse during childhood, adulthood, or both (cluster 8 was removed from subsequent analyses due to violations of the proportional hazards assumption across all exposures). Individuals who experienced abuse during both stages of life (whether sexual or physical) exhibited the greatest risk of hospitalisations (Table 1, lower panels). After accounting for age, sex, education, and ethnic/racial origin, and compared with individuals without a history of physical and sexual abuse, these participants had a more than 2-fold increase in the risk of mental and behavioural disorders (clusters 6 and 7; range of adjusted HRs 2.12–3.37). The risk of metabolic, haematologic, and respiratory diseases was approximately 1.5-fold (clusters 1, 2 and 4; HR range 1.46–1.83), whereas the risk of inflammatory diseases was about 1.2-fold (clusters 3 and 5; HR range 1.20–1.29). The separation in the crude hazard curves between participants exposed to physical abuse in both childhood and adulthood and those not exposed continued the entire follow-up for six of the seven outcome clusters (Supplement, p 25; violations of the proportional hazards assumption were observed for the cluster of musculoskeletal disorders, which we therefore omitted from subsequent analyses). We further tested potential time-dependent effects of these associations by adding an interaction term between abuse and the logarithm of the follow-up time to each model (Supplement, p 26). While the results showed no significant interactions for six of the seven associations (p > 0.05), the association of abuse with the “diabetes, anaemia, and hypertension” cluster slightly attenuated during the second half of the follow-up (Years 0–2.5: 1.67, 1.32–2.12; years +2.5: 1.43, 1.07–1.92; p for interaction 0.021). In addition, we found no statistically significant interactions between exposure to physical/sexual abuse during childhood and adulthood and either frailty or (pre-) existing comorbidities (p > 0.05), suggesting that these factors did not significantly alter the associations of abuse with disease risk (Supplement, p 14; note that analyses on clusters 6 and cluster 7 could not be estimated due to the rarity of outcome events). The associations between lifecourse exposure to physical/sexual abuse and disease clusters were also robust to adjustment for competing risk of death (Supplement, p 27). Comparison of exposure-outcome associations across all abuse measures suggests that most associations were driven by exposure to physical and sexual abuse in early life.

**Table 1:** Multivariable-adjusted associations of childhood abuse, adulthood abuse, and repeated abuse across both life stages with 7 disease clusters (UK Biobank).

	Outcome						
	Cluster 1:	Cluster 2:	Cluster 3:	Cluster 4:	Cluster 5:	Cluster 6:	Cluster 7:
	Diabetes, anaemia, hypertension	Sleep disorders, hospital-treated obesity	Inflammatory bowel diseases, bacterial infections, viral infections	Chronic obstructive bronchitis, influenza and pneumonia, asthma	Soft tissue disorders, osteoarthritis, rheumatoid arthritis, sciatica	Poisonings, mood disorders, neurotic disorders	Alcoholic liver disease, disorders due to substance abuse
**Exposure: Childhood abuse**^a^							
Abused [N (cases)/N (total)]	505/29,455	54/29,802	770/28,649	459/29,615	1529/26,149	72/29,769	17/30,089
Not abused [N (cases)/N (total)]	1211/89,978	111/91,312	2026/88,145	1193/90,650	4386/81,404	155/91,385	38/91,873
**Adjusted**^b^**relative risk**							
Hazard ratio (95% CI) after adjustment for confounders	1.36 (1.22–1.51)	1.40 (1.01–1.95)	1.22 (1.13–1.33)	1.31 (1.17–1.46)	1.17 (1.10–1.24)	1.44 (1.08–1.90)	1.28 (0.72–2.28)
**Incidence**							
Abused (per 100,000 person-years)	374.34	39.30	590.70	338.40	1308.11	52.46	12.26
Not abused (per 100,000 person-years)	293.86	26.37	505.16	287.35	1205.36	36.79	8.97
**Adjusted**^b^**absolute risk**							
Risk difference (95% CI) after adjustment for confounders	105.79 (64.65–149.87)	10.55 (0.26–25.05)	111.14 (65.67–166.7)	89.08 (48.85–132.18)	204.91 (120.54–289.29)	16.19 (2.94–33.11)	2.51 (−2.51 to 11.48)
**Exposure: Adulthood abuse**^c^							
Abused [N (cases)/N (total)]	261/17,908	33/18,127	421/17,358	253/17,973	922/15,960	48/18,036	14/18,276
Not abused [N (cases)/N (total)]	1455/101,525	132/102,987	2375/99,436	1399/102,292	4993/91,593	179/103,118	41/103,686
**Adjusted**^b^**relative risk**							
Hazard ratio (95% CI) after adjustment for confounders	Not estimated^d^	1.53 (1.04–2.27)	1.09 (0.98–1.21)	1.26 (1.10–1.44)	Not estimated^d^	1.45 (1.05–2.00)	2.58 (1.38–4.81)
**Incidence**							
Abused (per 100,000 person-years)	–	39.49	533.05	307.35	–	57.73	16.62
Not abused (per 100,000 person-years)	–	27.80	524.94	298.61	–	37.65	8.58
**Adjusted**^b^**absolute risk**							
Risk difference (95% CI) after adjustment for confounders	–	14.74 (1.11–35.31)	47.24 (−10.5 to 110.24)	77.64 (29.86–131.39)	–	16.94 (1.88–37.65)	13.55 (3.26–32.68)
**Exposure: Childhood and adulthood abuse**^e^							
Abused [N (cases)/N (total)]	130/7449	15/7527	185/7183	109/7469	400/6539	27/7477	8/7611
Not abused [N (cases)/N (total)]	1080/79,519	93/80,712	1790/77,970	1049/80,146	3864/71,983	134/80,826	32/81,208
**Adjusted**^b^**relative risk**							
Hazard ratio (95% CI) after adjustment for confounders	1.54 (1.28–1.86)	1.83 (1.05–3.20)	1.24 (1.07–1.45)	1.46 (1.20–1.79)	Not estimated^d^	2.12 (1.39–3.23)	3.37 (1.52–7.45)
**Incidence**							
Abused (per 100,000 person-years)	381.05	43.23	566.05	318.64	–	78.33	22.80
Not abused (per 100,000 person-years)	296.54	24.99	504.56	285.78	–	35.96	8.55
**Adjusted**^b^**absolute risk**							
Risk difference (95% CI) after adjustment for confounders	160.13 (83.03–255.03)	20.75 (1.25–54.99)	121.09 (35.32–227.05)	131.46 (57.16–225.76)	–	40.28 (14.03–80.2)	20.26 (4.44–55.13)

a Comparison between participants with vs without childhood abuse.

b Adjusted for age, sex ethnic/racial origin, and education.

c Comparison between participants with vs without adulthood abuse.

d The proportional hazards assumptions not met.

e Comparison between participants with vs without childhood and adulthood abuse.

In Table 1, we also show the multivariable-adjusted absolute risk for the 7 disease clusters, along with the absolute risk differences between individuals with and without a history of abuse. The highest hospitalisation rates were found in individuals who had been exposed to physical and sexual abuse during both childhood and adulthood. In this group, the greatest absolute risk difference was observed for metabolic and haematologic conditions (rate per 100,000 person-years rate 381, risk difference per 100 000 person-years compared with non-exposed individuals 160). Diseases of the respiratory system ranked second (rate 319, risk difference 131). The smallest absolute risk differences were observed for mental and behavioural disorders (rate 78, risk difference 40) and disorders related to substance abuse (rate 23, risk difference 20).

**Fig. 6:**
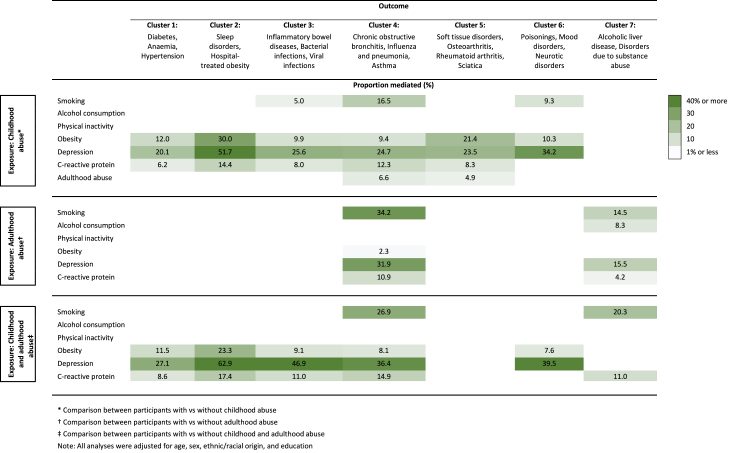
**Mediators of the associations of childhood abuse, adulthood abuse, and repeated abuse across both life stages with mental and physical health outcomes (UK Biobank)**.

Causal mediation analyses suggested that self-reported depressive symptoms, obesity, and inflammation acted as mediators in most abuse-disease cluster associations, with baseline depressive symptoms accounting for 16%–63%, obesity for 2%–30%, and inflammation for 4%–17% of these associations (Fig. 6). In sensitivity analyses, we further explored whether depressive symptoms remained a significant mediator after additionally adjusting effect estimates for childhood physical and emotional neglect. While there was a slight attenuation in the total proportion mediated by depressive symptoms, they continued to act as a strong intermediatory factor (Supplement, p 28). Furthermore, we examined the mediating role of inflammation in a subgroup of individuals without obesity. The results indicate that elevated levels of CRP remained a mediator in the majority of exposure-outcome associations, albeit with comparatively smaller effects (Supplement, p 29). Smoking appeared to be most relevant for behavioural, circulatory, and respiratory disease clusters, with the total proportion mediated ranging from 5% to 34%. By contrast, excessive self-reported alcohol consumption was a relatively weak mediator, and no significant indirect effects were found for physical activity.

## Discussion

The findings from this study suggest that exposure to physical and sexual abuse during early and later stages of life is associated with an increase in hospitalisations related to 22 non-overlapping diseases. The greatest risk of hospitalisations was found in individuals who had experienced abuse both in childhood and adulthood. These individuals had an over 2-fold risk of hospital-treated mental and behavioural disorders, an approximately 1.5 times increased risk of metabolic, haematologic, and respiratory diseases, and an around 1.2 higher risk of inflammatory diseases, compared with their non-exposed counterparts. The greatest absolute risk difference between individuals with versus those without a history of abuse was found for metabolic and haematologic conditions, and diseases of the respiratory system. The majority of abuse-disease cluster relationships were, at least in part, attributable to elevated depressive symptoms, obesity, and inflammation, whereas smoking acted as a dominant mediator primarily for behavioural, circulatory, and respiratory conditions.

To our knowledge, this is the first study to employ an outcome-wide approach within a single analytical setting to examine the associations between early and later life physical and sexual abuse and a wide array of mental and physical health conditions. Prior meta-analytic reviews in this field have reported considerable methodological heterogeneity across studies and insufficient adjustment for potential confounding factors.^2^^,^^5^^,^^16^^,^^17^ These limitations have hindered meaningful comparison of the effects of abuse in relation to different disease outcomes. In contrast, our approach enabled us to compare the relative importance of each abuse–disease relationship using homogenous measurements, accounting for various important confounders, and investigating the role of psychological, behavioural, and biological factors.

Overall, our findings reveal a generalised pattern of association between abuse and poor health, adding to evidence from previous studies. The elevated risk of mental and behavioural disorders, substance abuse-related disorders, respiratory diseases, and diseases of the circulatory system in people with a history of childhood or adulthood abuse confirm previous meta-analytic investigations and large-scale population-based studies.^4^^,^^5^^,^^15^, ^16^, ^17^^,^^30^^,^^31^ Much of this work had relied on self-report measures of ill health rather than linkage data to national hospital records as we have used here. Furthermore, previous studies on the health impacts of childhood abuse have primarily focussed on single or cumulative measures of various adverse childhood experiences,^5^ but not on individuals who had been exposed to physical and sexual abuse during both early and later stages of life.

In our study, self-reported depressive symptoms emerged as the strongest intermediatory factor in most abuse-disease relationships. This aligns with prior studies reporting an increased risk of mood disorders in abuse survivors,^16^^,^^32^ and a link between depression and various endocrine, respiratory and musculoskeletal disorders.^33^ Obesity was a strong mediator of the association between abuse and diabetes, anaemia, and hypertension, confirming previous studies on the relationship between abuse and obesity,^5^ and between obesity and morbidity.^34^ Systemic inflammation was identified as an additional underlying factor, particularly in relation to respiratory diseases, inflammatory conditions, sleep problems, and hospital-treated obesity. These results expand upon previous research linking childhood and adulthood abuse with immune system disturbances,^30^^,^^35^^,^^36^ and systemic inflammation with various physical illnesses.^37^ Notably, the magnitude of mediation exerted by systemic inflammation was considerably reduced in individuals without obesity, suggesting that obesity may be an underlying factor driving the mediating effect of inflammation. Other untested possible biological mechanisms include stress-induced accelerated ageing (*e.g.*, telomere attrition, accumulation of senescent cells, DNA methylation changes), corticosteroid-related stress pathways; structural changes in the brain (*e.g.*, reduced cortical volume); and alterations in monoamine transmitter secretion.^2^^,^^23^^,^^38^

Our findings also support a potential mediating role of behavioural factors. Smoking mediated the associations between abuse and circulatory, respiratory, and behavioural conditions. While the findings on the role of self-reported alcohol use were inconclusive, participants who had been subjected to both childhood and adulthood abuse had a 3-fold higher risk of hospitalisations from alcoholic liver disease, substance abuse disorders, and poisonings. These data concur with prior observations indicating that individuals with a history of abuse are more likely to engage in adverse health behaviours, which in turn, can elevate the risk of health complications later in life.^5^^,^^39^

Our findings are plausible. The strong associations with mental and behavioural disorders and substance abuse-related disorders as well as the mediating role of behavioural factors in abuse–diseases associations highlight the important role of psychological factors in excess ill health among victims of physical and sexual abuse. Psychological stress, in addition to the damage caused by substance abuse and other health risk behaviours, may also contribute to an elevated risk of metabolic disorders and communicative diseases because long-term stress is related to reduced self-care, impairs metabolism by increasing lipid accrual, lipolysis, and blood glucose concentration, and induces immune dysfunction and low-grade inflammation.^38^

Interpretation of our findings requires consideration of various limitations. Our study is based on observational data, thus precluding the possibility of inferring causation. The accuracy of self-reported abuse may be influenced by biases in memory, repression of past experiences, mental health problems such as depression, and an individual's comfort level with sharing sensitive information, possibly leading to under- or overestimation of associations.^40^^,^^41^ This applies particularly to adverse childhood experiences reported during adulthood. While the primary aim of this study was to examine the association of lifecourse exposure to physical and sexual abuse with physical and mental health conditions, future studies are needed to examine the health ramifications of other common types of adversities, such as emotional and physical neglect.^1^^,^^42^ Since the covariates and exposure to abuse were measured at the same time, establishing a temporal order between abuse and the measured biological, psychological, and behavioural factors is not possible. Conclusions about the temporal sequence between abuse and disease onset may be hampered owing to missing data on undiagnosed diseases and disease diagnoses made in primary care. Utilising hospitalisation records to ascertain disease outcomes misses milder health problems. Additionally, the prevalence of undiagnosed diseases tends to be higher in those with disadvantaged backgrounds. These issues may, if anything, contribute to an underestimation of the strength of abuse-disease associations. Furthermore, while the Finnish Public Sector study achieved a participation rate of 80.7% among invitees, the UK Biobank cohort had a much lower baseline (5.5%) and follow-up (46.4%) response rate. Loss to follow-up can introduce bias, leading to the potential for overestimation or underestimation of risk factor–outcome associations. Due to the selection process associated with low response rates, participants in the UK Biobank tend to have more favourable risk factor levels and are generally healthier than UK's general population.^43^ We sought to evaluate the magnitude of potential bias by examining whether factors that may contribute to attrition (frailty, comorbidities, competing risk of death) modified abuse-disease relationships in UK Biobank, as well as by repeating the main analyses in an independent cohort of Finnish adults with a higher response rate. While no clear evidence was found for a modifying role of these factors or survival bias, and our main findings were replicated in FPS, skewed risk-factor outcome associations cannot be ruled out. Lastly, with the large majority of participants in the present analyses having a White ethnic/racial background, the generalisability of our results to minority groups is unknown.

In conclusion, we showed that possible repercussions of both childhood and adulthood physical and sexual abuse pervade mental and physical health domains, with widespread associations across multiple organ systems. Policy makers and clinicians should be aware of these potential long-lasting health consequences for victims, irrespective of age. Our findings highlight the need for an intensified focus on abuse prevention within public health strategies. These include, for example, educational initiatives, reinforcement of routine screening procedures, and early detection systems for abuse in primary and secondary care. Interventions that target modifiable intermediary factors that lie on the pathway between exposure to abuse in early and later life and subsequent physical illness, such as mental health problems and lifestyle factors, may also be beneficial.

## Contributors

PF and MK generated the idea for the paper. All authors contributed significantly to the conception, design, and analysis or interpretation of data. PF wrote the first draft of the manuscript and other authors were involved in revising it critically for intellectual context. PF, JP, and MK had full access to the anonymized individual-participant data from all constituent studies and take responsibility for the integrity of the data and the accuracy of the data analyses. The corresponding author attests that all listed authors meet authorship criteria and that no others meeting the criteria have been omitted. The final submission of this paper was approved by all authors. PF, JP, and MK have verified the underlying data.

## Data sharing statement

Syntax for data analysis is provided in the appendix (pp 15–16). Our data protection agreements with the participating cohort studies do not allow us to share individual-level data from these studies to third parties. Pre-existing individual-level data access policies for each of the participating cohort studies specify that research data requests can be submitted to each steering committee; these will be promptly reviewed for confidentiality, data protection issues, or intellectual property restrictions and will not unreasonably be refused. Researchers registered with UK Biobank can apply for access to the database by completing an application. This must include a summary of the research plan, data-fields required, any new data or variables that will be generated, and payment to cover the incremental costs of servicing an application (https://www.ukbiobank.ac.uk/enable-your-research/apply-for-access).

## Declaration of interests

All authors report no competing interests.
