# Supplementary material for: Impact of physical and sexual abuse on risk of hospitalisations for physical and mental illnesses: insights from two large prospective cohort studies
Source: Lancet Reg Health Eur. 2024 Mar 12;40:100883. doi: 10.1016/j.lanepe.2024.100883 (PMC10944261; doi:10.1016/j.lanepe.2024.100883)
Supplement: Supplement Figures and Tables [file mmc1.docx]

Data supplement for Frank et al. Physical and sexual abuse and risk of hospitalisations for physical and mental illnesses: Analysis of two large prospective cohort studies

CONTENTS

[eTable 1. Test of the Cox proportional hazards assumptions (childhood physical abuse) 3](#_Toc158806300)

[eTable 2. Test of the Cox proportional hazards assumptions (childhood sexual abuse) 5](#_Toc158806301)

[eTable 3. Test of the Cox proportional hazards assumptions (adulthood physical abuse) 7](#_Toc158806302)

[eTable 4. Test of the Cox proportional hazards assumptions (adulthood sexual abuse) 9](#_Toc158806303)

[eFigure 1: Log-log plot childhood physical/sexual abuse: log(follow-up) versus log(-log(survival)) for 7 main disease clusters 11](#_Toc158806304)

[eFigure 2. Log-log plot adulthood physical/sexual abuse: log(follow-up) versus log(-log(survival)) for 7 main disease clusters 12](#_Toc158806305)

[eFigure 3. Log-log plot childhood and adulthood physical/sexual abuse: log(follow-up) versus log(-log(survival)) for 7 main disease clusters 13](#_Toc158806306)

[eTable 5. Statistical significance of the interaction terms between childhood and adulthood abuse and frailty, and between childhood and adulthood abuse and comorbidity on disease clusters (UK Biobank) 14](#_Toc158806307)

[eMethods. Statistical Code STATA. Loop for Cox proportional hazards regression analyses (example, UK Biobank) 15](#_Toc158806308)

[eTable 6. Baseline (2016–17) characteristics in the UK Biobank study 17](#_Toc158806309)

[eTable 7. Baseline characteristics in the Finnish Public Sector study 18](#_Toc158806310)

[eTable 8. Differences in 2006/10 baseline characteristics between the analytical and baseline sample 19](#_Toc158806311)

[eTable 9. Age and sex-adjusted associations of physical and sexual abuse during childhood and adulthood with mental and physical health conditions in UK Biobank 20](#_Toc158806312)

[eTable 10. Age and sex-adjusted associations of physical and sexual abuse during childhood and adulthood with mental and physical health conditions in the Finnish Public Sector study 23](#_Toc158806313)

[eFigure 4. Cumulative incidence of morbidity in individuals with versus without repeated physical or sexual abuse during childhood and adulthood (UK Biobank) 25](#_Toc158806314)

[eTable 11. Statistical significance of the interaction term between childhood and adulthood abuse and logarithmically transformed follow-up time (UK Biobank) 26](#_Toc158806315)

[eTable 12. Associations of abuse in childhood and adulthood with disease clusters before and after adjustment for the competing risk of death (Fine and Gray analysis in UK Biobank) 27](#_Toc158806316)

[eTable 13. Depression as a mediator of the associations of childhood abuse, adulthood abuse, and repeated abuse across both life stages with mental and physical health outcomes after adjustment for age, sex, ethnic/racial origin, education, physical neglect, and emotional neglect 28](#_Toc158806317)

[eTable 14. Inflammation as a mediator of the associations of childhood abuse, adulthood abuse, and repeated abuse across both life stages with mental and physical health outcomes after adjustment for age, sex, ethnic/racial origin, education, and obesity 29](#_Toc158806318)

[eTable 15. Age- and sex-adjusted associations of physical and emotional neglect during childhood with mental and physical health conditions in UK Biobank 30](#_Toc158806319)

[eTable 16. Associations of physical and sexual abuse during childhood with mental and physical health conditions in UK Biobank – hazard ratios and 95% CIs adjusted for age, sex, emotional neglect, and physical neglect 32](#_Toc158806320)

[eTable 17. Associations of physical abuse during childhood with mental and physical health conditions in UK Biobank – hazard ratios and 95% CIs adjusted for age, sex, and sexual abuse 34](#_Toc158806321)

[eTable 18. Frequencies and cases per abuse measure (childhood) 36](#_Toc158806322)

[eTable 19. Frequencies and cases per abuse measure (adulthood) 40](#_Toc158806323)

eTable 1. Test of the Cox proportional hazards assumptions (childhood physical abuse)

| **Disease outcome** | **N (total)** | **N (cases)** | **ChiSq (df=3)** | **ProbChiSq** |
| --- | --- | --- | --- | --- |
| **Infections** | 153454 | 2831 | 9.8047 | 0.0203 |
| Bacterial infections | 154596 | 2394 | 5.7332 | 0.1253 |
| Viral infections | 155918 | 346 | 2.9303 | 0.4025 |
| **Cancer** | 142284 | 9108 | 7.1827 | 0.0663 |
| Colorectal cancer | 155732 | 831 | 4.7801 | 0.1886 |
| Lung cancer | 156768 | 469 | 2.8964 | 0.4079 |
| Melanoma | 152005 | 3028 | 3.6386 | 0.3032 |
| Breast cancer (women) | 83740 | 1248 | 6.7974 | 0.0334 |
| Prostate cancer (men) | 64632 | 1407 | 9.946 | 0.0069 |
| Kidney cancer | 156698 | 213 | 3.849 | 0.2782 |
| Brain cancer | 156929 | 121 | 3.3557 | 0.34 |
| Leukaemia, lymphoma | 156007 | 647 | 1.2723 | 0.7357 |
| **Diseases of the blood** | 153189 | 2163 | 1.7225 | 0.6319 |
| Anaemia | 153998 | 1879 | 0.7093 | 0.871 |
| **Endocrine diseases** | 154455 | 1276 | 15.9393 | 0.0012 |
| Diabetes | 156604 | 150 | 0.4785 | 0.9236 |
| Obesity requiring hospital treatment | 156804 | 52 | 6.896 | 0.0753 |
| **Mental and behavioural disorders** | 155897 | 386 | 9.3445 | 0.025 |
| Dementia | 156933 | 78 | 2.9853 | 0.3939 |
| Disorders due to substance abuse | 156767 | 46 | 3.3417 | 0.3419 |
| Mood disorders | 156539 | 75 | 4.48 | 0.2141 |
| Neurotic disorders | 156637 | 106 | 0.5923 | 0.8982 |
| Psychotic disorders | 156862 | 33 | 5.1983 | 0.1578 |
| **Diseases of the nervous system** | 149136 | 2564 | 16.2064 | 0.001 |
| Parkinson disease | 156912 | 79 | 4.3724 | 0.224 |
| Multiple sclerosis | 156740 | 38 | 0.5406 | 0.9099 |
| Epilepsy | 156741 | 71 | 6.0085 | 0.1112 |
| Headaches | 156338 | 270 | 2.6805 | 0.4436 |
| TIA | 156316 | 384 | 3.834 | 0.28 |
| Sleep disorders | 155776 | 183 | 1.7808 | 0.6191 |
| **Diseases of the eye** | 144408 | 8341 | 0.8667 | 0.8335 |
| **Diseases of the ear** | 154867 | 551 | 7.5248 | 0.0569 |
| **Diseases of the circulatory system** | 136008 | 7865 | 1.4134 | 0.7024 |
| Hypertension | 156591 | 292 | 1.3918 | 0.7075 |
| Ischemic heart diseases | 150438 | 2845 | 0.2039 | 0.977 |
| Angina pectoris | 154796 | 604 | 0.84 | 0.8399 |
| Myocardial infarction | 154892 | 1150 | 2.0158 | 0.5691 |
| Pulmonary embolism | 156141 | 594 | 0.6633 | 0.8818 |
| Arrhythmias | 153474 | 1924 | 1.0433 | 0.7908 |
| Heart failure | 156702 | 424 | 1.6495 | 0.6482 |
| Stroke | 155808 | 1057 | 4.5722 | 0.2059 |
| Cerebrovascular diseases | 155540 | 1214 | 1.8448 | 0.6052 |
| Intracerebral haemorrhage | 156832 | 155 | 1.6125 | 0.6566 |
| Cerebral infarction | 156284 | 777 | 2.8843 | 0.4098 |
| Arteriosclerosis | 156807 | 92 | 5.5608 | 0.135 |
| Deep vein thrombosis | 156044 | 470 | 1.9181 | 0.5896 |
| **Diseases of the respiratory system** | 148591 | 3942 | 1.0895 | 0.7796 |
| Influenza and pneumonia | 155246 | 1777 | 3.2641 | 0.3527 |
| Chronic obstructive bronchitis | 156529 | 398 | 7.6538 | 0.0537 |
| Asthma | 156305 | 198 | 0.6175 | 0.8924 |

*eTable 1 continued from previous page*

| **Disease outcome** | **N (total)** | **N (cases)** | **ChiSq (df=3)** | **ProbChiSq** |
| --- | --- | --- | --- | --- |
| **Diseases of the digestive system** | 112236 | 15754 | 4.7952 | 0.1874 |
| Appendicitis | 155832 | 280 | 1.1766 | 0.7586 |
| Inflammatory bowel disease | 152709 | 1111 | 8.4596 | 0.0374 |
| Diseases of liver | 156499 | 269 | 2.5052 | 0.4744 |
| Alcoholic liver disease | 156913 | 30 | 1.7542 | 0.625 |
| Pancreatitis | 156478 | 200 | 5.1805 | 0.159 |
| **Diseases of the skin** | 145990 | 3579 | 6.106 | 0.1066 |
| Skin infections and eczema | 154015 | 1071 | 4.194 | 0.2413 |
| **Diseases of the musculoskeletal system** | 128407 | 10423 | 0.4951 | 0.92 |
| Rheumatoid arthritis and related disorders | 155685 | 500 | 9.6931 | 0.0214 |
| Gout | 156864 | 57 | 1.3167 | 0.7252 |
| Osteoarthritis | 147609 | 4777 | 5.5762 | 0.1341 |
| Sciatica | 154712 | 652 | 3.1551 | 0.3683 |
| Back pain | 154022 | 1044 | 2.0617 | 0.5597 |
| Soft tissue disorders | 147042 | 2945 | 2.8619 | 0.4134 |
| **Diseases of the genitourinary system** | 127824 | 5940 | 25.7661 | 0 |
| Renal failure | 156598 | 463 | 3.5665 | 0.3122 |
| **Pregnancy complications** | 154277 | 2 | 3.9952 | 0.1357 |
| Spontaneous abortion | 156342 | 1 | . | . |
| Hypertension in pregnancy | 156611 | 1 | . | . |
| Diabetes in pregnancy | 156914 | . | . | . |
| **Miscellaneous** |  |  |  |  |
| Circulatory and respiratory symptoms | 145013 | 4053 | 2.4006 | 0.4935 |
| Digestive and abdominal symptoms | 143205 | 4876 | 2.8139 | 0.4212 |
| Injury | 144725 | 4667 | 11.6852 | 0.0085 |
| Poisoning | 156162 | 166 | 5.9981 | 0.1117 |
| Road accidents | 156958 | 2 | 0.7575 | 0.3841 |
| Falls | 156957 | 15 | 3.1673 | 0.3665 |

eTable 2. Test of the Cox proportional hazards assumptions (childhood sexual abuse)

| **Disease outcome** | **N (total)** | **N (cases)** | **ChiSq (df=3)** | **ProbChiSq** |
| --- | --- | --- | --- | --- |
| **Infections** | 152007 | 2794 | 7.7421 | 0.0517 |
| Bacterial infections | 153133 | 2368 | 5.1656 | 0.1601 |
| Viral infections | 154442 | 337 | 2.1357 | 0.5447 |
| **Cancer** | 140929 | 9032 | 8.3229 | 0.0398 |
| Colorectal cancer | 154252 | 822 | 4.0919 | 0.2517 |
| Lung cancer | 155277 | 463 | 1.5541 | 0.6698 |
| Melanoma | 150564 | 3012 | 3.4866 | 0.3225 |
| Breast cancer (women) | 82611 | 1226 | 6.2925 | 0.0430 |
| Prostate cancer (men) | 64348 | 1404 | 10.7505 | 0.0046 |
| Kidney cancer | 155205 | 209 | 4.0637 | 0.2547 |
| Brain cancer | 155436 | 118 | 3.5504 | 0.3143 |
| Leukaemia, lymphoma | 154521 | 643 | 0.98 | 0.8061 |
| **Diseases of the blood** | 151738 | 2137 | 0.9797 | 0.8062 |
| Anaemia | 152529 | 1857 | 0.217 | 0.9748 |
| **Endocrine diseases** | 152994 | 1263 | 20.0286 | 0.0002 |
| Diabetes | 155112 | 149 | 1.7928 | 0.6165 |
| Obesity requiring hospital treatment | 155311 | 50 | 7.7505 | 0.0515 |
| **Mental and behavioural disorders** | 154429 | 383 | 9.5093 | 0.0232 |
| Dementia | 155439 | 76 | 2.7041 | 0.4395 |
| Disorders due to substance abuse | 155276 | 46 | 2.8224 | 0.4198 |
| Mood disorders | 155060 | 74 | 4.2433 | 0.2364 |
| Neurotic disorders | 155148 | 106 | 0.6453 | 0.886 |
| Psychotic disorders | 155374 | 32 | 3.1228 | 0.3731 |
| **Diseases of the nervous system** | 147749 | 2519 | 17.1722 | 0.0007 |
| Parkinson disease | 155419 | 79 | 4.4909 | 0.2131 |
| Multiple sclerosis | 155246 | 36 | 1.9407 | 0.5848 |
| Epilepsy | 155250 | 70 | 3.1099 | 0.375 |
| Headaches | 154854 | 263 | 1.3431 | 0.7189 |
| TIA | 154826 | 380 | 4.1581 | 0.2449 |
| Sleep disorders | 154298 | 182 | 0.9587 | 0.8113 |
| **Diseases of the eye** | 143036 | 8255 | 11.3582 | 0.0099 |
| **Diseases of the ear** | 153399 | 542 | 6.6074 | 0.0855 |
| **Diseases of the circulatory system** | 134730 | 7786 | 0.6785 | 0.8783 |
| Hypertension | 155101 | 286 | 0.6068 | 0.8949 |
| Ischemic heart diseases | 149009 | 2820 | 0.4449 | 0.9308 |
| Angina pectoris | 153341 | 598 | 1.6687 | 0.6439 |
| Myocardial infarction | 153406 | 1141 | 1.8468 | 0.6048 |
| Pulmonary embolism | 154655 | 588 | 1.0982 | 0.7775 |
| Arrhythmias | 151997 | 1897 | 2.1604 | 0.5398 |
| Heart failure | 155210 | 419 | 3.0306 | 0.3869 |
| Stroke | 154335 | 1050 | 4.9645 | 0.1744 |
| Cerebrovascular diseases | 154074 | 1206 | 1.9682 | 0.579 |
| Intracerebral haemorrhage | 155338 | 151 | 1.5246 | 0.6766 |
| Cerebral infarction | 154802 | 774 | 2.9383 | 0.4012 |
| Arteriosclerosis | 155314 | 91 | 0.576 | 0.9019 |
| Deep vein thrombosis | 154555 | 466 | 2.0962 | 0.5527 |
| **Diseases of the respiratory system** | 147203 | 3898 | 0.9231 | 0.8199 |
| Influenza and pneumonia | 153775 | 1758 | 3.3758 | 0.3372 |
| Chronic obstructive bronchitis | 155043 | 389 | 8.855 | 0.0313 |
| Asthma | 154826 | 196 | 0.2606 | 0.9673 |

*eTable 2 continued from previous page*

| **Disease outcome** | **N (total)** | **N (cases)** | **ChiSq (df=3)** | **ProbChiSq** |
| --- | --- | --- | --- | --- |
| **Diseases of the digestive system** | 111265 | 15606 | 5.4187 | 0.1436 |
| Appendicitis | 154356 | 273 | 4.6263 | 0.2013 |
| Inflammatory bowel disease | 151276 | 1101 | 15.5671 | 0.0014 |
| Diseases of liver | 155010 | 263 | 3.0126 | 0.3897 |
| Alcoholic liver disease | 155419 | 30 | 6.4193 | 0.0929 |
| Pancreatitis | 154988 | 196 | 4.4364 | 0.218 |
| **Diseases of the skin** | 144627 | 3546 | 5.6571 | 0.1295 |
| Skin infections and eczema | 152564 | 1061 | 4.4065 | 0.2208 |
| **Diseases of the musculoskeletal system** | 127242 | 10297 | 3.0843 | 0.3788 |
| Rheumatoid arthritis and related disorders | 154210 | 490 | 10.7404 | 0.0132 |
| Gout | 155370 | 55 | 3.7244 | 0.2928 |
| Osteoarthritis | 146222 | 4720 | 6.5683 | 0.087 |
| Sciatica | 153235 | 642 | 4.2029 | 0.2404 |
| Back pain | 152573 | 1028 | 1.782 | 0.6189 |
| Soft tissue disorders | 145659 | 2897 | 2.1326 | 0.5454 |
| **Diseases of the genitourinary system** | 126702 | 5873 | 25.9098 | 0 |
| Renal failure | 155105 | 456 | 4.1057 | 0.2503 |
| **Pregnancy complications** | 152813 | 2 | 1.7255 | 0.189 |
| Spontaneous abortion | 154856 | 1 | . | . |
| Hypertension in pregnancy | 155116 | 1 | . | . |
| Diabetes in pregnancy | 155420 | . | . | . |
| **Miscellaneous** |  |  |  |  |
| Circulatory and respiratory symptoms | 143665 | 4002 | 0.9062 | 0.8239 |
| Digestive and abdominal symptoms | 141898 | 4808 | 3.0691 | 0.3811 |
| Injury | 143329 | 4633 | 13.2618 | 0.0041 |
| Poisoning | 154687 | 163 | 11.3926 | 0.0098 |
| Road accidents | 155464 | 2 | 0.7521 | 0.6866 |
| Falls | 155463 | 15 | 2.6805 | 0.2618 |

eTable 3. Test of the Cox proportional hazards assumptions (adulthood physical abuse)

| **Disease outcome** | **N (total)** | **N (cases)** | **ChiSq (df=3)** | **ProbChiSq** |
| --- | --- | --- | --- | --- |
| **Infections** | 153323 | 2825 | 10.8995 | 0.0123 |
| Bacterial infections | 154465 | 2389 | 6.3143 | 0.0973 |
| Viral infections | 155784 | 345 | 2.0666 | 0.5587 |
| **Cancer** | 142165 | 9108 | 9.0899 | 0.0281 |
| Colorectal cancer | 155604 | 831 | 6.6872 | 0.0826 |
| Lung cancer | 156636 | 469 | 5.1699 | 0.1598 |
| Melanoma | 151875 | 3027 | 5.0545 | 0.1679 |
| Breast cancer (women) | 83625 | 1248 | 6.4298 | 0.0402 |
| Prostate cancer (men) | 64620 | 1407 | 9.6102 | 0.0082 |
| Kidney cancer | 156565 | 213 | 5.6663 | 0.129 |
| Brain cancer | 156796 | 121 | 3.5477 | 0.3146 |
| Leukaemia, lymphoma | 155874 | 650 | 2.7183 | 0.4371 |
| **Diseases of the blood** | 153056 | 2158 | 5.4182 | 0.1436 |
| Anaemia | 153864 | 1874 | 6.1931 | 0.1026 |
| **Endocrine diseases** | 154326 | 1271 | 18.3114 | 0.0004 |
| Diabetes | 156468 | 151 | 1.4431 | 0.6955 |
| Obesity requiring hospital treatment | 156671 | 51 | 8.8734 | 0.031 |
| **Mental and behavioural disorders** | 155777 | 385 | 11.2447 | 0.0105 |
| Dementia | 156800 | 78 | 4.8266 | 0.1849 |
| Disorders due to substance abuse | 156635 | 46 | 2.8573 | 0.4142 |
| Mood disorders | 156413 | 74 | 3.6692 | 0.2995 |
| Neurotic disorders | 156504 | 106 | 2.7782 | 0.4271 |
| Psychotic disorders | 156733 | 33 | 5.8847 | 0.1174 |
| **Diseases of the nervous system** | 149017 | 2557 | 15.7992 | 0.0012 |
| Parkinson disease | 156779 | 76 | 5.5753 | 0.1342 |
| Multiple sclerosis | 156607 | 38 | 0.3906 | 0.9422 |
| Epilepsy | 156609 | 70 | 5.1485 | 0.1612 |
| Headaches | 156206 | 270 | 4.0783 | 0.2531 |
| TIA | 156184 | 386 | 6.0377 | 0.1098 |
| Sleep disorders | 155645 | 183 | 1.9301 | 0.587 |
| **Diseases of the eye** | 144280 | 8306 | 3.4139 | 0.3321 |
| **Diseases of the ear** | 154736 | 551 | 6.2119 | 0.1017 |
| **Diseases of the circulatory system** | 135889 | 7853 | 4.2533 | 0.2354 |
| Hypertension | 156458 | 291 | 0.7348 | 0.865 |
| Ischemic heart diseases | 150304 | 2840 | 1.8924 | 0.595 |
| Angina pectoris | 154662 | 605 | 3.1106 | 0.3749 |
| Myocardial infarction | 154755 | 1147 | 2.1852 | 0.5349 |
| Pulmonary embolism | 156008 | 593 | 0.6739 | 0.8793 |
| Arrhythmias | 153341 | 1921 | 2.2779 | 0.5168 |
| Heart failure | 156569 | 423 | 2.1723 | 0.5374 |
| Stroke | 155675 | 1058 | 4.1898 | 0.2417 |
| Cerebrovascular diseases | 155407 | 1216 | 0.9353 | 0.8169 |
| Intracerebral haemorrhage | 156698 | 155 | 1.0983 | 0.7775 |
| Cerebral infarction | 156153 | 778 | 2.3757 | 0.4982 |
| Arteriosclerosis | 156674 | 91 | 1.7972 | 0.6156 |
| Deep vein thrombosis | 155911 | 470 | 1.876 | 0.5985 |
| **Diseases of the respiratory system** | 148464 | 3939 | 4.4968 | 0.2126 |
| Influenza and pneumonia | 155112 | 1776 | 11.342 | 0.01 |
| Chronic obstructive bronchitis | 156397 | 397 | 8.4302 | 0.0379 |
| Asthma | 156170 | 198 | 0.5914 | 0.8984 |

*eTable 3 continued from previous page*

| **Disease outcome** | **N (total)** | **N (cases)** | **ChiSq (df=3)** | **ProbChiSq** |
| --- | --- | --- | --- | --- |
| **Diseases of the digestive system** | 112158 | 15731 | 5.3228 | 0.1496 |
| Appendicitis | 155702 | 278 | 3.9952 | 0.262 |
| Inflammatory bowel disease | 152581 | 1109 | 4.8776 | 0.181 |
| Diseases of liver | 156367 | 267 | 3.1916 | 0.363 |
| Alcoholic liver disease | 156780 | 30 | 1.293 | 0.7308 |
| Pancreatitis | 156342 | 200 | 4.3412 | 0.2269 |
| **Diseases of the skin** | 145861 | 3576 | 7.493 | 0.0577 |
| Skin infections and eczema | 153882 | 1070 | 4.2818 | 0.2326 |
| **Diseases of the musculoskeletal system** | 128309 | 10414 | 0.8706 | 0.8325 |
| Rheumatoid arthritis and related disorders | 155555 | 499 | 8.4024 | 0.0384 |
| Gout | 156731 | 57 | 2.3505 | 0.5029 |
| Osteoarthritis | 147496 | 4774 | 5.8966 | 0.1167 |
| Sciatica | 154572 | 649 | 2.9145 | 0.405 |
| Back pain | 153890 | 1042 | 0.769 | 0.8569 |
| Soft tissue disorders | 146913 | 2939 | 1.4609 | 0.6913 |
| **Diseases of the genitourinary system** | 127725 | 5938 | 27.4516 | 0 |
| Renal failure | 156463 | 464 | 3.3798 | 0.3367 |
| **Pregnancy complications** | 154145 | 2 | 3.2296 | 0.3576 |
| Spontaneous abortion | 156209 | 1 | . | . |
| Hypertension in pregnancy | 156476 | 1 | . | . |
| Diabetes in pregnancy | 156781 | . | . | . |
| **Miscellaneous** |  |  |  |  |
| Circulatory and respiratory symptoms | 144873 | 4050 | 1.259 | 0.7389 |
| Digestive and abdominal symptoms | 143097 | 4869 | 2.2709 | 0.5181 |
| Injury | 144607 | 4663 | 12.8182 | 0.005 |
| Poisoning | 156033 | 166 | 5.4604 | 0.141 |
| Road accidents | 156825 | 2 | 0.7519 | 0.6866 |
| Falls | 156824 | 15 | 2.8521 | 0.415 |

eTable 4. Test of the Cox proportional hazards assumptions (adulthood sexual abuse)

| **Disease outcome** | **N (total)** | **N (cases)** | **ChiSq (df=3)** | **ProbChiSq** |
| --- | --- | --- | --- | --- |
| **Infections** | 153298 | 2825 | 9.6892 | 0.0214 |
| Bacterial infections | 154441 | 2389 | 6.0351 | 0.1099 |
| Viral infections | 155759 | 344 | 2.3153 | 0.5096 |
| **Cancer** | 142148 | 9100 | 8.5705 | 0.0356 |
| Colorectal cancer | 155583 | 830 | 3.5596 | 0.3131 |
| Lung cancer | 156615 | 469 | 4.3352 | 0.2275 |
| Melanoma | 151853 | 3023 | 3.8369 | 0.2796 |
| Breast cancer (women) | 83578 | 1244 | 5.6795 | 0.0584 |
| Prostate cancer (men) | 64643 | 1408 | 10.6516 | 0.0049 |
| Kidney cancer | 156542 | 213 | 4.5221 | 0.2103 |
| Brain cancer | 156772 | 120 | 3.8162 | 0.282 |
| Leukaemia, lymphoma | 155851 | 648 | 2.1576 | 0.5404 |
| **Diseases of the blood** | 153036 | 2156 | 3.0163 | 0.3891 |
| Anaemia | 153843 | 1875 | 1.8586 | 0.6023 |
| **Endocrine diseases** | 154299 | 1270 | 17.1633 | 0.0007 |
| Diabetes | 156443 | 151 | 2.2032 | 0.5313 |
| Obesity requiring hospital treatment | 156648 | 51 | 8.4921 | 0.0369 |
| **Mental and behavioural disorders** | 155746 | 383 | 12.4063 | 0.0061 |
| Dementia | 156776 | 78 | 2.9422 | 0.4006 |
| Disorders due to substance abuse | 156610 | 46 | 5.9804 | 0.1126 |
| Mood disorders | 156384 | 73 | 3.5266 | 0.3173 |
| Neurotic disorders | 156481 | 106 | 5.1324 | 0.1624 |
| Psychotic disorders | 156708 | 32 | 3.7935 | 0.2846 |
| **Diseases of the nervous system** | 148986 | 2558 | 16.5983 | 0.0009 |
| Parkinson disease | 156755 | 78 | 5.7087 | 0.1267 |
| Multiple sclerosis | 156583 | 38 | 1.0574 | 0.7874 |
| Epilepsy | 156585 | 70 | 3.6722 | 0.2991 |
| Headaches | 156182 | 270 | 3.5551 | 0.3137 |
| TIA | 156158 | 385 | 3.4555 | 0.3266 |
| Sleep disorders | 155621 | 184 | 1.4247 | 0.6997 |
| **Diseases of the eye** | 144252 | 8306 | 3.3995 | 0.334 |
| **Diseases of the ear** | 154710 | 552 | 7.1285 | 0.0679 |
| **Diseases of the circulatory system** | 135859 | 7853 | 2.5883 | 0.4595 |
| Hypertension | 156434 | 291 | 1.0339 | 0.7931 |
| Ischemic heart diseases | 150281 | 2842 | 5.8087 | 0.1213 |
| Angina pectoris | 154641 | 606 | 1.6018 | 0.659 |
| Myocardial infarction | 154732 | 1148 | 1.5429 | 0.6724 |
| Pulmonary embolism | 155983 | 593 | 1.293 | 0.7308 |
| Arrhythmias | 153316 | 1919 | 4.5283 | 0.2098 |
| Heart failure | 156545 | 423 | 1.3887 | 0.7082 |
| Stroke | 155650 | 1059 | 3.6576 | 0.3009 |
| Cerebrovascular diseases | 155382 | 1217 | 1.9867 | 0.5752 |
| Intracerebral haemorrhage | 156674 | 154 | 4.9657 | 0.1743 |
| Cerebral infarction | 156128 | 780 | 2.678 | 0.444 |
| Arteriosclerosis | 156650 | 91 | 1.5729 | 0.6655 |
| Deep vein thrombosis | 155888 | 470 | 1.0333 | 0.7932 |
| **Diseases of the respiratory system** | 148443 | 3939 | 1.4878 | 0.6851 |
| Influenza and pneumonia | 155089 | 1775 | 3.5153 | 0.3188 |
| Chronic obstructive bronchitis | 156376 | 398 | 8.3263 | 0.0397 |
| Asthma | 156148 | 198 | 1.0183 | 0.7968 |

*eTable 4 continued from previous page*

| **Disease outcome** | **N (total)** | **N (cases)** | **ChiSq (df=3)** | **ProbChiSq** |
| --- | --- | --- | --- | --- |
| **Diseases of the digestive system** | 112137 | 15729 | 4.9005 | 0.1792 |
| Appendicitis | 155681 | 279 | 1.9474 | 0.5834 |
| Inflammatory bowel disease | 152556 | 1110 | 4.9601 | 0.1747 |
| Diseases of liver | 156343 | 268 | 3.0125 | 0.3897 |
| Alcoholic liver disease | 156756 | 30 | 0.7575 | 0.8596 |
| Pancreatitis | 156317 | 199 | 4.6749 | 0.1972 |
| **Diseases of the skin** | 145841 | 3579 | 9.8824 | 0.0196 |
| Skin infections and eczema | 153856 | 1070 | 4.8445 | 0.1835 |
| **Diseases of the musculoskeletal system** | 128289 | 10417 | 1.5607 | 0.6683 |
| Rheumatoid arthritis and related disorders | 155534 | 499 | 7.7471 | 0.0515 |
| Gout | 156707 | 57 | 2.7176 | 0.4372 |
| Osteoarthritis | 147461 | 4776 | 5.9173 | 0.1157 |
| Sciatica | 154553 | 651 | 3.1373 | 0.3709 |
| Back pain | 153870 | 1044 | 1.7038 | 0.6361 |
| Soft tissue disorders | 146896 | 2947 | 1.4373 | 0.6968 |
| **Diseases of the genitourinary system** | 127712 | 5937 | 31.1538 | 0 |
| Renal failure | 156440 | 463 | 4.6979 | 0.1953 |
| **Pregnancy complications** | 154130 | 2 | 3.2194 | 0.359 |
| Spontaneous abortion | 156188 | 1 | . | . |
| Hypertension in pregnancy | 156454 | 1 | . | . |
| Diabetes in pregnancy | 156757 | . | . | . |
| **Miscellaneous** |  |  |  |  |
| Circulatory and respiratory symptoms | 144855 | 4041 | 0.6756 | 0.8789 |
| Digestive and abdominal symptoms | 143073 | 4865 | 2.1141 | 0.5491 |
| Injury | 144583 | 4659 | 14.6875 | 0.0021 |
| Poisoning | 156007 | 166 | 7.652 | 0.0538 |
| Road accidents | 156801 | 2 | 0.7557 | 0.6853 |
| Falls | 156800 | 15 | 3.0529 | 0.3835 |

eFigure 1: Log-log plot childhood physical/sexual abuse: log(follow-up) versus log(-log(survival)) for 7 main disease clusters

**
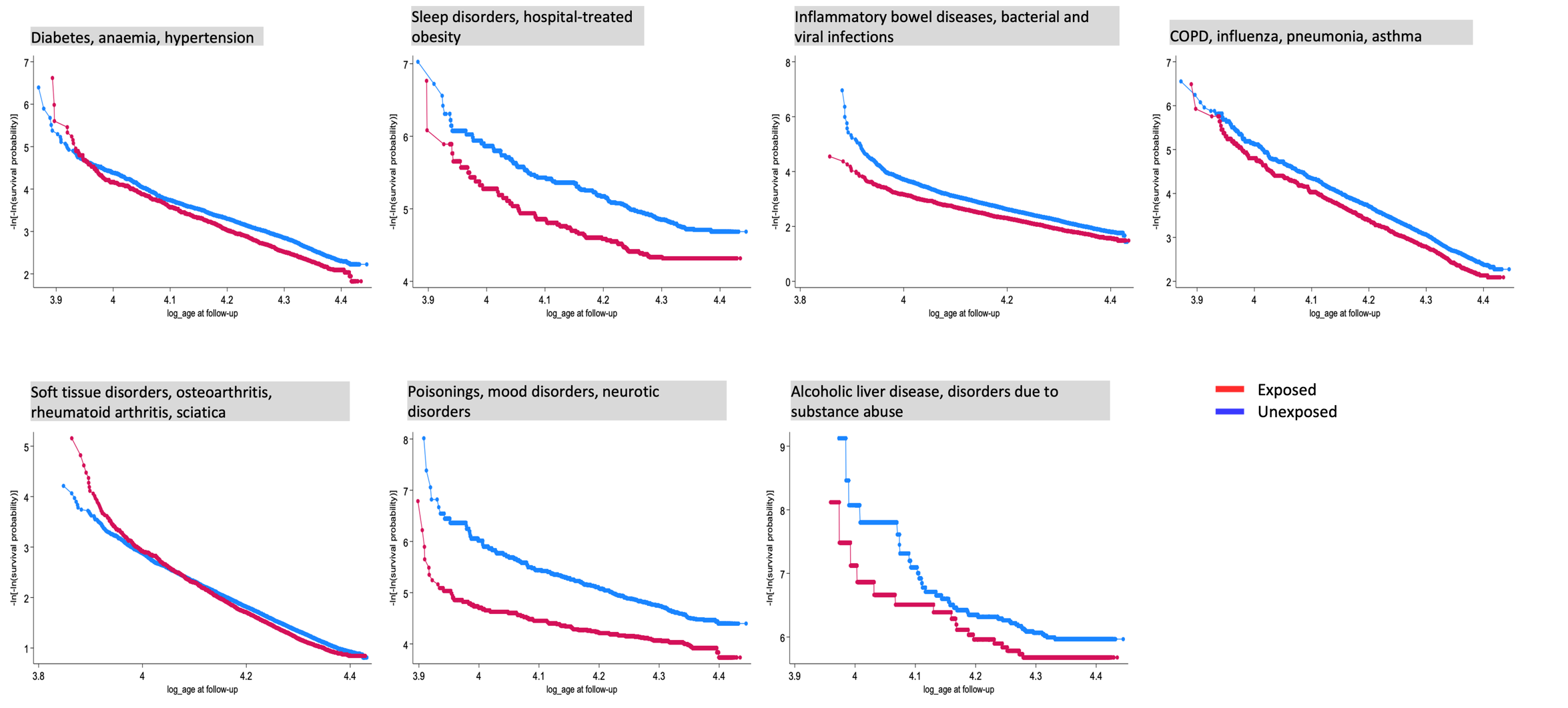
**

eFigure 2. Log-log plot adulthood physical/sexual abuse: log(follow-up) versus log(-log(survival)) for 7 main disease clusters

**
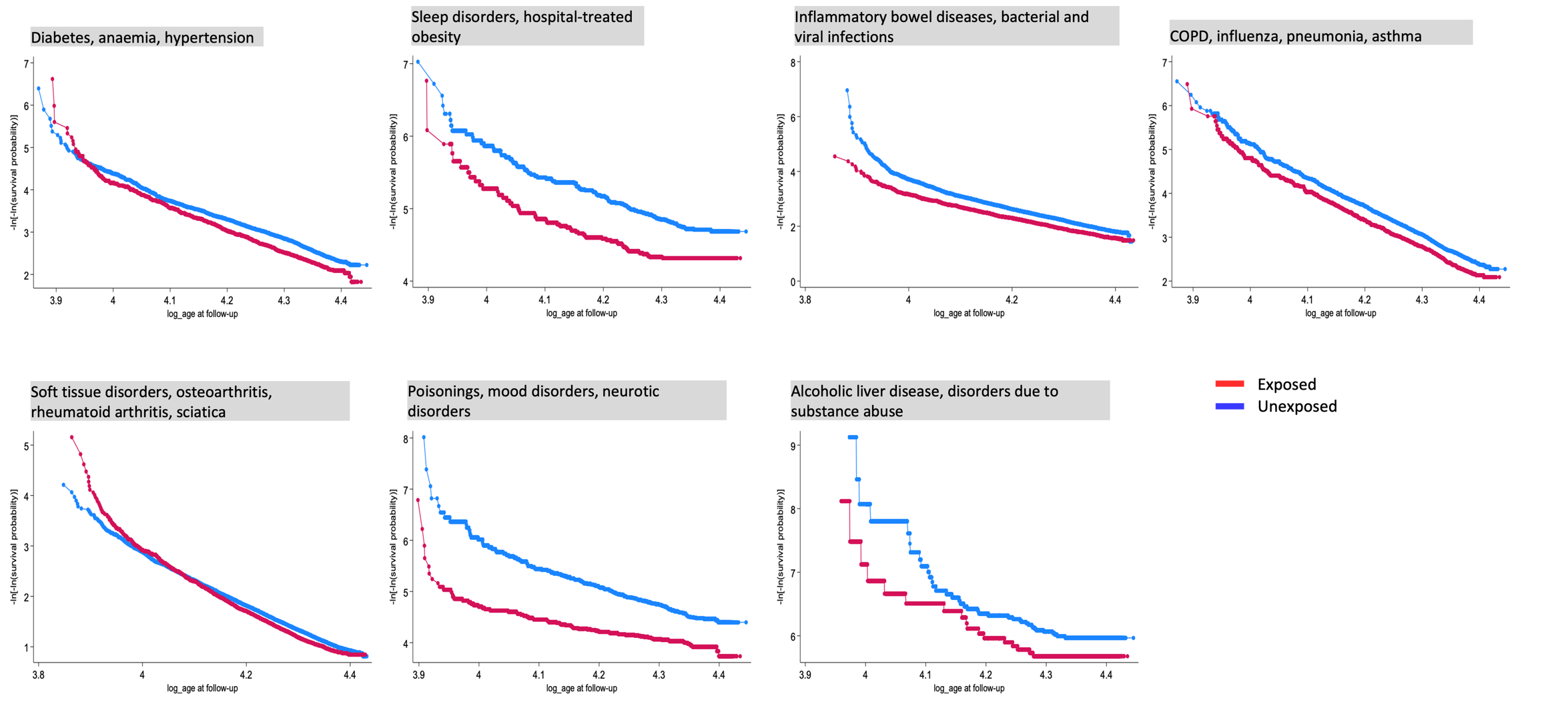
**

eFigure 3. Log-log plot childhood and adulthood physical/sexual abuse: log(follow-up) versus log(-log(survival)) for 7 main disease clusters

**
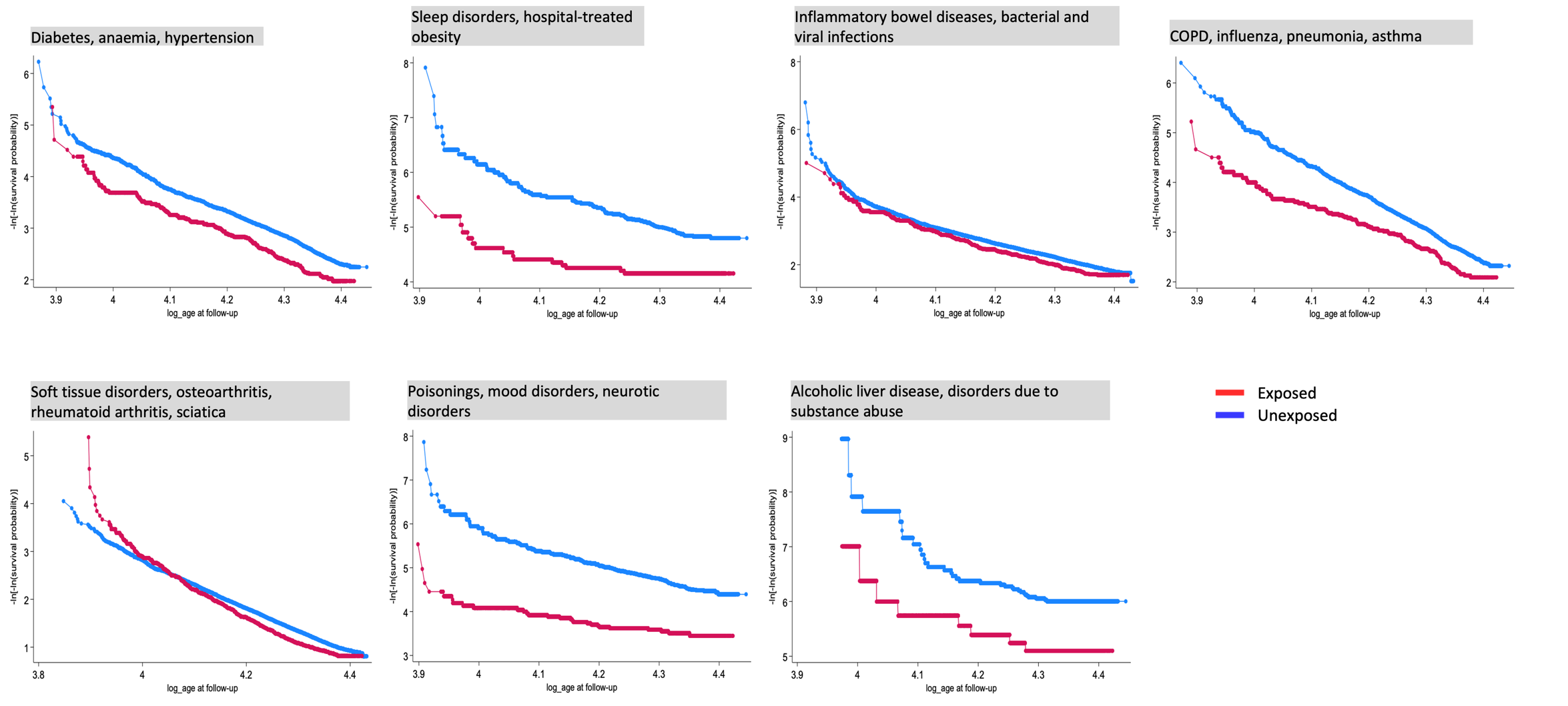
**

eTable 5. Statistical significance of the interaction terms between childhood and adulthood abuse and frailty, and between childhood and adulthood abuse and comorbidity on disease clusters (UK Biobank)

Utilising data from the UK Biobank study, we performed separate Cox proportional hazards regression analyses to investigate the modulation of associations between lifecourse physical/sexual abuse exposure and health outcomes by comorbidities and frailty, incorporating interaction terms for both. Two binary variables were created to denote past or present comorbidities (yes, no) and frailty (yes, no). Our measurement of comorbidities before or at baseline was based on linkage data to national hospital and mortality registries. Individual diseases and disease categories were coded according to the International Classification of Diseases 10th Revision (ICD-10). Participants were classified as having a comorbidity if they were hospitalised due to at least one of the following conditions: cardiometabolic conditions, such as diabetes, obesity, anaemia, and hypertension; inflammatory disorders such as inflammatory bowel syndrome, bacterial infections, and viral infections; respiratory disorders such as chronic obstructive pulmonary disease (COPD), influenza, asthma, and pneumonia; as well as behavioural and mental health disorders, including sleep disorders, neurotic disorders, mood disorders, substance-related disorders, and alcoholic liver disease. For each health outcome, participants with the disease at or before baseline, as ascertained from the linked health registry data, were excluded from the analysis of incident hospital-treated conditions at follow-up. Frailty was measured via the Fried frailty phenotype (Fried LP, Tangen CM, Walston J, Newman AB, Hirsch C, Gottdiener J, Seeman T, Tracy R, Kop WJ, Burke G, McBurnie MA. Frailty in older adults: Evidence for a phenotype. J Gerontol A Biol Sci Med Sci 56: M146–M156) – a widely used measure that incorporates the following aspects of frailty: unintentional weight loss (“Compared with one year ago, has your weight changed?”; yes=1, no=0), weakness (i.e., grip strength, sex and body-mass index adjusted cut-offs taken from Fried and colleague; yes=1, no=0), exhaustion (“Over the past two weeks, how often have you felt tired or had little energy?”; more than half the days or nearly every day=1, other = 0), slow walking speed (“How would you describe your usual walking pace?”; slow=1, other=0), and physical inactivity (none/light physical activity once per week or less=1, medium/heavy physical activity, or light physical activity more than once per week=0). We found no statistically significant interaction effects between exposure to physical/sexual abuse and either frailty or the broad category of comorbidities (p>0.05), indicating that these health-related factors might not substantially alter the relationship between sexual/physical abuse and disease risk in our sample. The analyses of interactions between physical/sexual abuse and frailty were successfully performed for all disease clusters (cluster 5 was not examined because the proportionality assumption was not met). Analyses related to interactions with comorbidity were successful for all but two clusters. This is because interaction terms with comorbidities with cluster 6 (poisonings, mood disorders, neurotic disorders) and cluster 7 (alcoholic liver disease, disorders due to substance abuse) could not be estimated due to the rarity of disease cases.

eMethods. Statistical Code STATA. Loop for Cox proportional hazards regression analyses (example, UK Biobank)

capture program drop prog_getpred

program define prog_getpred

args row outcome title

matrix B=r(table)

local b=round(B[1,2],.01)

local lci=round(B[5,2],.01)

local uci=round(B[6,2],.01)

local p = B[4,2]

local string "`b' (`lci' to `uci')"

putexcel A`row'="`outcome'"

putexcel B`row'="`title'"

putexcel C`row'="`b'"

putexcel D`row'="`lci'"

putexcel E`row'="`uci'"

putexcel F`row'="`p'"

putexcel G`row'="`string'"

end

putexcel set "UKB_ICD10_physical_abuse_childhood.xlsx", modify sheet("Sheet1", replace)

local covariates i.w1sex w1age_MQ

local outcomes Infections_ICD10 ///

BacInfections_ICD10 ///

ViralInfections_ICD10 ///

Cancer_ICD10 ///

ColCancer_ICD10 ///

LungCancer_ICD10 ///

MelanomaCancer_ICD10 ///

BreastCancer_ICD10 ///

ProstateCancer_ICD10 ///

KidneyCancer_ICD10 ///

BrainCancer_ICD10 ///

LeukaemiaCancer_ICD10 ///

Blood_ICD10 ///

Anaemias_ICD10 ///

Endocrine_ICD10 ///

Diabetes_ICD10 ///

Obesity_ICD10 ///

Mental_ICD10 ///

Dementia_ICD10 ///

Substance_ICD10 ///

Mood_ICD10 ///

Neurotic_ICD10 ///

Psychotic_ICD10 ///

NS_ICD10 ///

Parkinson_ICD10 ///

MS_ICD10 ///

Epilepsy_ICD10 ///

Headaches_ICD10 ///

TIA_ICD10 ///

Sleep_ICD10 ///

Eye_ICD10 ///

Ear_ICD10 ///

Circulatory_ICD10 ///

Hypertension_ICD10 ///

IschemicH_ICD10 ///

Angina_ICD10 ///

Myocardial_ICD10 ///

Pulmonary_ICD10 ///

Arrhythmias_ICD10 ///

HeartFailure_ICD10 ///

Stroke_ICD10 ///

Cerebrovascular_ICD10 ///

Intracerebral_ICD10 ///

CerebralInfarction_ICD10 ///

Arteriosclerosis_ICD10 ///

DeepVein_ICD10 ///

Respiratory_ICD10 ///

Influenzia_ICD10 ///

Obstructive_ICD10 ///

Asthma_ICD10 ///

Digestive_ICD10 ///

Appendicitis_ICD10 ///

IBS_ICD10 ///

Liver_ICD10 ///

AlcLiver_ICD10 ///

Pancreatitis_ICD10 ///

Skin_ICD10 ///

SkinInfect_ICD10 ///

Musculoskeletal_ICD10 ///

Rheumatoid_ICD10 ///

Gout_ICD10 ///

Osteoarthritis_ICD10 ///

Sciatica_ICD10 ///

BackPain_ICD10 ///

SoftTissue_ICD10 ///

Genitourinary_ICD10 ///

RenalFailure_ICD10 ///

Pregnancy_ICD10 ///

Abortion_ICD10 ///

HypertensionPreg_ICD10 ///

DiabetesPreg_ICD10 ///

CircRespSymp_ICD10 ///

DigestiveSymp_ICD10 ///

Injury_ICD10 ///

Poisoning_ICD10 ///

RoadA_ICD10 ///

Falls_ICD10

local exposure i.ace_physcialA

local row=0

foreach outcome of local outcomes {

local row=`row'+1

stset date_`outcome', failure(`outcome'==1) id(n_eid) origin(birth_day) enter(date_mentalQ) scale(365.25)

stcox `exposure' `covariates'

prog_getpred `row' `outcome' }

import excel using "UKB_ICD10_physical_abuse_childhood.xlsx", sheet(Sheet1) clear

reshape wide C, i(B) j(A) string

rename C* *

rename B covariates

eTable 6. Baseline (2016–17) characteristics in the UK Biobank study

|  | **UK Biobank (All)** | **No exposure to physical or sexual abuse across the lifecourse** | **Exposure to physical or sexual abuse across the lifecourse** |  |
| --- | --- | --- | --- | --- |
|  |  |  |  | **p-value** |
|  |  |  |  |  |
| Participants (n, %) | 122 123 (100%) | 81 280 (66.6%) | 40 843 (33.4%) |  |
| Age (years, SD) | 63.3 (7.6) | 63.9 (7.7) | 62.2 (7.7) | p < 0.01 |
| Sex (%) |  |  |  |  |
| Men | 45.6 (55,686) | 48.3 (39,292) | 40.1% (16 394) |  |
| Women | 54.4 (66,437) | 51.7 (41,988) | 59.9% (24 449) | p < 0.01 |
| Depression (%) |  |  |  |  |
| No | 94.4 (115,338) | 96.2 (78,191) | 91.0 (37,147) |  |
| Yes | 5.6 (6,785) | 3.8 (3,089) | 9.0 (3,696) | p < 0.01 |
| Ethnic/racial origin (%) |  |  |  |  |
| White | 97.2 (118,757) | 97.9 (79,572) | 95.9 (39,185) |  |
| Non-white | 2.8 (3,366) | 2.1 (1,708) | 4.1 (1,658) | p < 0.01 |
| Education (%, n) |  |  |  |  |
| None/Elementary | 6.1 (7,385) | 6.2 (5,014) | 5.8 (2,371) |  |
| Secondary | 46.3 (56,585) | 45.8 (37,206) | 47.4 (19,379) |  |
| Tertiary | 47.6 (58,153) | 48.0 (39,060) | 46.8 (19,093) | p < 0.01 |
| Smoking (%, n) |  |  |  |  |
| Never | 57.4 (70,119) | 60.7 (49,347) | 50.9 (20,772) |  |
| Previous | 35.4 (43,226) | 33.3 (27,074) | 39.5 (16,152) |  |
| Current | 7.2 (8,778) | 6.0 (4,859) | 9.6 (3,919) | p < 0.01 |
| Alcohol (%, n) |  |  |  |  |
| None | 13.9 (16,965) | 13.0 (10,532) | 15.7 (6,433) |  |
| Low | 35.5 (43,341) | 35.3 (28,691) | 35.9 (14,650) |  |
| Moderate | 26.7 (32,561) | 27.4 (22,283) | 25.2 (10,278) |  |
| High | 23.9 (29,256) | 24.3 (19,774) | 23.2 (9,482) | p < 0.01 |
| Physically inactive (%, n) |  |  |  |  |
| Yes | 46.4 (56,699) | 46.3 (37,593) | 46.8 (19,106) |  |
| No | 53.6 (65,424) | 53.7 (43,687) | 53.2 (21,737) | p = 0.08 |
| logC-reactive protein (mg/L) | 0.18 (0.003) | 0.15 (0.004) | 0.22 (0.005) | p < 0.01 |

eTable 7. Baseline characteristics in the Finnish Public Sector study

|  | **Finnish Public Sector study (All)** | **No exposure to physical or sexual abuse across the lifecourse** | **Exposure to physical or sexual abuse across the lifecourse** |  |
| --- | --- | --- | --- | --- |
|  |  |  |  | **p-value** |
|  |  |  |  |  |
| Participants (n, %) | 85 929 (100%) | 710279 (82.9%) | 14 650 (17.1%) |  |
| Age (years, SD) | 45.4 (10.7) | 45.3 (10.8) | 45.8 (10.1) | p < 0.01 |
| Sex (%) |  |  |  |  |
| Men | 20.2 (17 385) | 21.1 (15 014) | 16.2 (2371) |  |
| Women | 79.8 (68 544) | 78.9 (56 265) | 83.8 (12 279) | p < 0.01 |
| Depression (%) |  |  |  |  |
| No | 87.0 (73 514) | 89.7 (62 823) | 73.9 (10 691) |  |
| Yes | 13.0 (10 996) | 10.3 (7226) | 26.1 (3770) | p < 0.01 |
| Ethnic/racial origin (%) | – | – | – | – |
| White | – | – | – | – |
| Non-white | – | – | – | – |
| Education (%, n) |  |  |  |  |
| None/Elementary | 9.1 (7808) | 9.2 (6570) | 8.5 (1238) |  |
| Secondary | 33.5 (28 761) | 32.7 (23 327) | 37.1 (5434) |  |
| Tertiary | 57.4 (49 359) | 58.1 (41 381) | 54.5 (7978) | p < 0.01 |
| Smoking (%, n) |  |  |  |  |
| Never | 65.7 (55 209) | 68.3 (47 661) | 52.8 (7548) |  |
| Previous | 17.8 (14 941) | 16.8 (11 707) | 22.6 (3234) |  |
| Current | 16.6 (13 946) | 14.9 (10 431) | 24.6 (3515) | p < 0.01 |
| Alcohol (%, n) |  |  |  |  |
| None | 15.4 (13 143) | 15.3 (10 869) | 15.6 (2274) |  |
| Low | 74.2 (63 520) | 75.1 (53 262) | 70.3 (10 258) |  |
| Moderate | 4.3 (3651) | 4.1 (2893) | 5.2 (758) |  |
| High | 6.1 (5248) | 5.6 (3947) | 8.9 (1301) | p < 0.01 |
| Physically inactive (%, n) |  |  |  |  |
| Yes | 79.7 (67 971) | 79.9 (56 492) | 79.0 (11 479) |  |
| No | 20.3 (17 315) | 20.2 (14 259) | 21.0 (3056) | p = 0.02 |
| logC-reactive protein (mg/L) | – | – | – | – |

eTable 8. Differences in 2006/10 baseline characteristics between the analytical and baseline sample

|  | **UK Biobank (All)** | **UK Biobank (Analytical sample)** |
| --- | --- | --- |
|  |  |  |
|  |  |  |
| Participants (n, %) | 459 441 (100%) | 122 123 (100%) |
| Age (years, SD) | 56.51 (8.1) | 55.7 (7.76) |
| Sex (%) |  |  |
| Men | 45.7 (209,896) | 45.6 (55,686) |
| Women | 54.3 (249,545) | 54.4 (66,437) |
| Ethnic/racial origin (%) |  |  |
| White | 94.9 (435,935) | 97.2 (118,757) |
| Non-white | 5.1 (23,506) | 2.8 (3,366) |
| Education (%, n) |  |  |
| None/Elementary | 17.2 (78,874) | 6.1 (7,385) |
| Secondary | 50.0 (229,847) | 46.3 (56,585) |
| Tertiary | 32.8 (150,720) | 47.6 (58,153) |
| Smoking (%, n) |  |  |
| Never | 54.7 (251,552) | 57.4 (70,119) |
| Previous | 34.8 (159,704) | 35.4 (43,226) |
| Current | 10.5 (48,185) | 7.2 (8,778) |
| Alcohol (%, n) |  |  |
| None | 19.3 (88,692) | 13.9 (16,965) |
| Low | 37.0 (169,858) | 35.5 (43,341) |
| Moderate | 23.2 (106,700) | 26.7 (32,561) |
| High | 20.5 (94,191) | 23.9 (29,256) |
| Physically inactive (%, n) |  |  |
| Yes | 45.7 (202,736) | 46.4 (56,699) |
| No | 54.3 (170,854) | 53.6 (65,424) |

eTable 9. Age and sex-adjusted associations of physical and sexual abuse during childhood and adulthood with mental and physical health conditions in UK Biobank

|  | | **Hazard ratio (95% CI)* by exposure (UKB)** | | | | | | | |
| --- | --- | --- | --- | --- | --- | --- | --- | --- | --- |
| **Disease outcome** | | **Childhood physical abuse** | | **Childhood sexual abuse** | | **Adulthood physical abuse** | | **Adulthood physical abuse** | |
| **Infections** | | 1.22 (1.11 to 1.33) | | 1.26 (1.12 to 1.43) | | 1.18 (1.05 to 1.31) | | 1.32 (1.13 to 1.54) | |
| Bacterial infections | | 1.25 (1.14 to 1.38) | | 1.30 (1.14 to 1.48) | | 1.17 (1.04 to 1.32) | | 1.30 (1.10 to 1.54) | |
| Viral infections | | 1.05 (0.80 to 1.38) | | 1.09 (0.75 to 1.57) | | 1.35 (1.01 to 1.80) | | 1.67 (1.15 to 2.42) | |
| **Cancer** | | 1.10 (1.05 to 1.16) | | 1.04 (0.96 to 1.12) | | 1.08 (1.01 to 1.15) | | 1.07 (0.97 to 1.18) | |
| Colorectal cancer | | 0.97 (0.81 to 1.16) | | 0.91 (0.70 to 1.18) | | 1.20 (0.97 to 1.47) | | 1.49 (1.12 to 1.98) | |
| Lung cancer | | 1.73 (1.41 to 2.13) | | 1.31 (0.97 to 1.77) | | 1.55 (1.2 to 2.00) | | 1.47 (1.01 to 2.15) | |
| Melanoma | | 1.07 (0.97 to 1.17) | | 1.00 (0.87 to 1.14) | | 1.03 (0.92 to 1.16) | | 0.90 (0.74 to 1.08) | |
| Breast cancer (women) | | 1.09 (0.94 to 1.26) | | 0.96 (0.80 to 1.15) | | 1.12 (0.97 to 1.30) | | 0.97 (0.80 to 1.18) | |
| Prostate cancer (men) | | 1.06 (0.93 to 1.20) | | 1.05 (0.84 to 1.30) | | 0.85 (0.69 to 1.05) | | 0.62 (0.28 to 1.39) | |
| Kidney cancer | | 1.58 (1.16 to 2.15) | | 1.48 (0.95 to 2.30) | | 0.77 (0.47 to 1.27) | | 0.93 (0.43 to 2.00) | |
| Brain cancer | | 1.07 (0.68 to 1.69) | | 0.83 (0.41 to 1.71) | | 1.03 (0.59 to 1.81) | | 1.28 (0.59 to 2.81) | |
| Leukaemia, lymphoma | | 1.11 (0.92 to 1.36) | | 1.05 (0.79 to 1.39) | | 1.21 (0.96 to 1.53) | | 1.29 (0.91 to 1.84) | |
| **Diseases of the blood** | | 1.24 (1.12 to 1.38) | | 1.44 (1.26 to 1.64) | | 1.13 (0.99 to 1.28) | | 1.25 (1.04 to 1.49) | |
| Anaemia | | 1.28 (1.15 to 1.43) | | 1.51 (1.32 to 1.74) | | 1.14 (1.00 to 1.31) | | 1.26 (1.04 to 1.53) | |
| **Endocrine diseases** | | 1.24 (1.08 to 1.41) | | 1.30 (1.08 to 1.55) | | 1.20 (1.02 to 1.41) | | 1.42 (1.14 to 1.76) | |
| Diabetes | | 0.89 (0.58 to 1.37) | | 1.18 (0.67 to 2.09) | | 1.48 (0.94 to 2.35) | | 2.20 (1.16 to 4.20) | |
| Obesity requiring hospital treatment | | 2.23 (1.26 to 3.93) | | 2.67 (1.39 to 5.13) | | 3.28 (1.86 to 5.78) | | 2.23 (1.07 to 4.64) | |
| **Mental and behavioural disorders** | | 1.28 (1.00 to 1.63) | | 1.55 (1.14 to 2.10) | | 1.38 (1.05 to 1.83) | | 2.40 (1.73 to 3.33) | |
| Dementia | | 1.23 (0.70 to 2.17) | | 0.86 (0.35 to 2.14) | | 0.51 (0.18 to 1.39) | | 1.03 (0.32 to 3.35) | |
| Disorders due to substance abuse | | 1.49 (0.78 to 2.85) | | 2.19 (0.97 to 4.93) | | 3.17 (1.65 to 6.09) | | 4.22 (1.66 to 10.76) | |
| Mood disorders | | 1.29 (0.75 to 2.22) | | 2.21 (1.23 to 3.97) | | 1.97 (1.15 to 3.37) | | 2.64 (1.39 to 4.99) | |
| Neurotic disorders | | 1.58 (1.02 to 2.45) | | 1.39 (0.78 to 2.50) | | 1.16 (0.68 to 1.99) | | 2.96 (1.72 to 5.09) | |
| Psychotic disorders | | 0.97 (0.40 to 2.36) | | 1.41 (0.49 to 4.05) | | 1.75 (0.75 to 4.09) | | 2.13 (0.72 to 6.29) | |
| **Diseases of the nervous system** | | 1.24 (1.13 to 1.36) | | 1.19 (1.05 to 1.36) | | 1.25 (1.11 to 1.39) | | 1.30 (1.11 to 1.52) | |
| Parkinson disease | | 1.39 (0.81 to 2.38) | | 0.86 (0.35 to 2.12) | | 0.69 (0.28 to 1.71) | | 0.74 (0.18 to 3.10) | |
| Multiple sclerosis | | 1.00 (0.46 to 2.18) | | 0.52 (0.12 to 2.18) | | 1.24 (0.54 to 2.83) | | 0.32 (0.04 to 2.36) | |
| Epilepsy | | 0.61 (0.30 to 1.22) | | 0.87 (0.35 to 2.17) | | 1.43 (0.74 to 2.74) | | 2.92 (1.34 to 6.39) | |
| Headaches | | 1.65 (1.26 to 2.17) | | 1.74 (1.24 to 2.43) | | 1.9 (1.43 to 2.52) | | 1.64 (1.11 to 2.41) | |
| TIA | | 1.06 (0.81 to 1.37) | | 1.11 (0.78 to 1.59) | | 0.99 (0.71 to 1.37) | | 1.13 (0.70 to 1.81) | |
| Sleep disorders | | 1.15 (0.81 to 1.63) | | 1.61 (1.03 to 2.52) | | 1.34 (0.88 to 2.02) | | 2.17 (1.27 to 3.72) | |
| **Diseases of the eye** | | 1.06 (1.00 to 1.12) | | 1.06 (0.98 to 1.14) | | 1.05 (0.98 to 1.12) | | 1.07 (0.97 to 1.18) | |
| **Diseases of the ear** | | 1.24 (1.01 to 1.52) | | 1.20 (0.91 to 1.59) | | 0.97 (0.75 to 1.26) | | 0.80 (0.53 to 1.20) | |

*eTable 9 continued from previous page*

| **Disease outcome** | **Childhood physical abuse** | **Childhood sexual abuse** | **Adulthood physical abuse** | **Adulthood physical abuse** |
| --- | --- | --- | --- | --- |
| **Diseases of the circulatory system** | 1.16 (1.10 to 1.23) | 1.27 (1.17 to 1.37) | 1.08 (1.01 to 1.16) | 1.14 (1.02 to 1.27) |
| Hypertension | 1.25 (0.94 to 1.66) | 1.64 (1.17 to 2.30) | 1.05 (0.75 to 1.49) | 1.16 (0.73 to 1.84) |
| Ischemic heart diseases | 1.16 (1.05 to 1.27) | 1.24 (1.09 to 1.41) | 1.08 (0.95 to 1.21) | 1.09 (0.89 to 1.33) |
| Angina pectoris | 1.15 (0.94 to 1.41) | 1.48 (1.15 to 1.91) | 1.25 (0.98 to 1.59) | 1.19 (0.82 to 1.72) |
| Myocardial infarction | 1.11 (0.96 to 1.29) | 1.24 (1.01 to 1.52) | 1.04 (0.86 to 1.27) | 1.00 (0.71 to 1.41) |
| Pulmonary embolism | 1.06 (0.86 to 1.30) | 1.31 (1. 00 to 1.71) | 0.89 (0.68 to 1.16) | 1.14 (0.79 to 1.64) |
| Arrhythmias | 1.14 (1.02 to 1.28) | 1.23 (1.05 to 1.44) | 1.11 (0.96 to 1.28) | 0.93 (0.72 to 1.18) |
| Heart failure | 0.95 (0.73 to 1.23) | 1.13 (0.80 to 1.61) | 0.98 (0.70 to 1.37) | 1.38 (0.85 to 2.25) |
| Stroke | 1.06 (0.90 to 1.24) | 1.14 (0.92 to 1.41) | 1.16 (0.96 to 1.41) | 1.04 (0.77 to 1.41) |
| Cerebrovascular diseases | 1.03 (0.89 to 1.19) | 1.14 (0.93 to 1.40) | 1.16 (0.97 to 1.39) | 1.14 (0.86 to 1.50) |
| Intracerebral haemorrhage | 1.06 (0.70 to 1.60) | 0.86 (0.45 to 1.63) | 0.89 (0.51 to 1.54) | 0.46 (0.15 to 1.47) |
| Cerebral infarction | .91 (0.75 to 1.11) | 1.08 (0.83 to 1.40) | 1.28 (1.03 to 1.59) | 1.09 (0.76 to 1.56) |
| Arteriosclerosis | .83 (0.47 to 1.47) | 1.08 (0.50 to 2.34) | 1.35 (0.71 to 2.55) | 1.09 (0.33 to 3.54) |
| Deep vein thrombosis | .98 (0.78 to 1.24) | 1.23 (0.90 to 1.68) | 0.83 (0.61 to 1.14) | 1.19 (0.79 to 1.79) |
| **Diseases of the respiratory system** | 1.32 (1.23 to 1.43) | 1.33 (1.20 to 1.48) | 1.34 (1.22 to 1.46) | 1.37 (1.20 to 1.56) |
| Influenza and pneumonia | 1.25 (1.12 to 1.41) | 1.31 (1.12 to 1.53) | 1.23 (1.07 to 1.41) | 1.55 (1.27 to 1.88) |
| Chronic obstructive bronchitis | 1.51 (1.19 to 1.91) | 1.47 (1.07 to 2.02) | 2.02 (1.57 to 2.60) | 2.10 (1.47 to 3.02) |
| Asthma | 1.47 (1.06 to 2.04) | 1.19 (0.75 to 1.87) | 1.08 (0.72 to 1.62) | 1.10 (0.63 to 1.91) |
| **Diseases of the digestive system** | 1.17 (1.13 to 1.22) | 1.25 (1.18 to 1.31) | 1.19 (1.13 to 1.24) | 1.20 (1.12 to 1.29) |
| Appendicitis | 1.13 (0.85 to 1.51) | 1.47 (1.02 to 2.11) | 0.87 (0.6 to 1.26) | 0.76 (0.43 to 1.34) |
| Inflammatory bowel disease | 1.29 (1.12 to 1.49) | 1.18 (0.97 to 1.43) | 1.05 (0.88 to 1.25) | 1.02 (0.80 to 1.31) |
| Diseases of liver | 1.09 (0.80 to 1.47) | 0.95 (0.61 to 1.49) | 1.39 (0.99 to 1.94) | 1.67 (1.07 to 2.63) |
| Alcoholic liver disease | 1.31 (0.58 to 2.96) | 2.14 (0.74 to 6.17) | 1.80 (0.68 to 4.76) | 5.18 (1.41 to 19.07) |
| Pancreatitis | 1.54 (1.12 to 2.13) | 0.92 (0.55 to 1.54) | 1.13 (0.75 to 1.69) | 0.75 (0.38 to 1.49) |
| **Diseases of the skin** | 1.05 (0.97 to 1.14) | 1.11 (0.99 to 1.24) | 1.16 (1.05 to 1.28) | 1.12 (0.97 to 1.30) |
| Skin infections and eczema | 1.06 (0.91 to 1.23) | 1.15 (0.93 to 1.42) | 1.32 (1.11 to 1.57) | 1.23 (0.95 to 1.60) |
| **Diseases of the musculoskeletal system** | 1.26 (1.20 to 1.32) | 1.15 (1.08 to 1.23) | 1.18 (1.12 to 1.25) | 1.10 (1.02 to 1.20) |
| Rheumatoid arthritis and related disorders | 1.16 (0.93 to 1.45) | 1.00 (0.73 to 1.35) | 1.31 (1.03 to 1.66) | 1.32 (0.96 to 1.82) |
| Gout | 1.20 (0.65 to 2.24) | 1.12 (0.40 to 3.10) | 1.20 (0.51 to 2.82) | 0.84 (0.11 to 6.30) |
| Osteoarthritis | 1.17 (1.09 to 1.26) | 1.01 (0.91 to 1.12) | 1.11 (1.02 to 1.21) | 1.12 (0.99 to 1.26) |
| Sciatica | 1.30 (1.08 to 1.56) | 1.35 (1.06 to 1.73) | 1.01 (0.80 to 1.27) | 1.35 (1.00 to 1.82) |
| Back pain | 1.69 (1.47 to 1.94) | 1.60 (1.33 to 1.92) | 1.41 (1.20 to 1.66) | 1.25 (0.99 to 1.59) |
| Soft tissue disorders | 1.26 (1.15 to 1.37) | 1.25 (1.11 to 1.42) | 1.16 (1.05 to 1.30) | 1.09 (0.93 to 1.28) |

*eTable 9 continued from previous page*

| **Disease outcome** | **Childhood physical abuse** | **Childhood sexual abuse** | **Adulthood physical abuse** | **Adulthood physical abuse** |
| --- | --- | --- | --- | --- |
| **Diseases of the genitourinary system** | 1.17 (1.10 to 1.25) | 1.18 (1.08 to 1.29) | 1.09 (1.01 to 1.18) | 1.10 (0.98 to 1.23) |
| Renal failure | 1.24 (0.99 to 1.55) | 1.53 (1.14 to 2.06) | 1.40 (1.08 to 1.84) | 1.64 (1.10 to 2.44) |
| **Miscellaneous** |  |  |  |  |
| Circulatory and respiratory symptoms | 1.19 (1.10 to 1.28) | 1.43 (1.29 to 1.58) | 1.28 (1.17 to 1.40) | 1.40 (1.23 to 1.58) |
| Digestive and abdominal symptoms | 1.23 (1.15 to 1.32) | 1.32 (1.21 to 1.45) | 1.23 (1.13 to 1.33) | 1.20 (1.07 to 1.34) |
| Injury | 1.09 (1.01 to 1.18) | 1.10 (0.99 to 1.21) | 1.11 (1.01 to 1.2) | 1.08 (0.95 to 1.22) |
| Poisoning | 1.24 (0.86 to 1.79) | 1.59 (1.01 to 2.50) | 1.58 (1.07 to 2.34) | 2.01 (1.23 to 3.29) |
| Road accidents |  |  |  |  |
| Falls | 0.76 (0.17 to 3.38) |  | 1.72 (0.38 to 7.75) | 3.14 (0.36 to 27.34) |

*Hazard ratio for abuse as predictor of disease adjusted for age and sex at baseline. Participants with the specific health condition at or before baseline were excluded from the present analyses.

eTable 10. Age and sex-adjusted associations of physical and sexual abuse during childhood and adulthood with mental and physical health conditions in the Finnish Public Sector study

| **Disease outcome** | **History of physical or sexual abuse across the lifecourse** |
| --- | --- |
| **Infections** | 1.33 (1.20 to 1.48) |
| Bacterial infections | 1.33 (1.19 to 1.48) |
| Viral infections | 1.36 (1.04 to 1.78) |
| **Cancer** | 1.04 (0.96 to 1.12) |
| Colorectal cancer | 1.02 (0.79 to 1.32) |
| Lung cancer | 1.30 (0.93 to 1.82) |
| Melanoma | 0.90 (0.76 to 1.06) |
| Breast cancer (women) | 1.15 (1.03 to 1.29) |
| Prostate cancer (men) | 1.06 (0.78 to 1.45) |
| Kidney cancer | 0.76 (0.43 to 1.36) |
| Brain cancer | 1.27 (0.76 to 2.14) |
| Leukaemia, lymphoma | 0.95 (0.74 to 1.22) |
| **Diseases of the blood** | 1.32 (1.06 to 1.64) |
| Anaemia | 1.40 (1.04 to 1.87) |
| **Endocrine diseases** | 1.21 (1.13 to 1.29) |
| Diabetes | 1.21 (1.12 to 1.31) |
| Obesity requiring hospital treatment | 1.87 (1.46 to 2.39) |
| **Mental and behavioural disorders** | 2.05 (1.82 to 2.31) |
| Dementia | 1.11 (0.84 to 1.45) |
| Disorders due to substance abuse | 2.30 (1.83 to 2.89) |
| Mood disorders | 2.37 (1.96 to 2.86) |
| Neurotic disorders | 2.44 (2.08 to 2.86) |
| Psychotic disorders | 2.21 (1.65 to 2.95) |
| **Diseases of the nervous system** | 1.26 (1.17 to 1.36) |
| Parkinson disease | 0.63 (0.41 to 0.99) |
| Multiple sclerosis | 0.78 (0.52 to 1.19) |
| Epilepsy | 1.26 (0.99 to 1.60) |
| Headaches | 1.31 (0.98 to 1.76) |
| TIA | 1.18 (0.96 to 1.46) |
| Sleep disorders | 1.57 (1.42 to 1.73) |
| **Diseases of the eye** | 1.04 (0.96 to 1.13) |
| **Diseases of the ear** | 1.11 (0.92 to 1.34) |
| **Diseases of the circulatory system** | 1.08 (1.01 to 1.15) |
| Hypertension | 1.12 (1.03 to 1.21) |
| Ischemic heart diseases | 1.14 (1.02 to 1.28) |
| Angina pectoris | 1.16 (0.96 to 1.38) |
| Myocardial infarction | 1.18 (0.96 to 1.44) |
| Pulmonary embolism | 1.14 (0.87 to 1.50) |
| Arrhythmias | 1.10 (1.01 to 1.21) |
| Heart failure | 1.14 (0.88 to 1.48) |
| Stroke | 1.25 (1.07 to 1.46) |
| Cerebrovascular diseases | 1.22 (1.03 to 1.45) |
| Intracerebral haemorrhage | 1.07 (0.68 to 1.68) |
| Cerebral infarction | 1.08 (0.87 to 1.34) |
| Arteriosclerosis | 1.29 (0.87 to 1.91) |
| Deep vein thrombosis | 0.94 (0.70 to 1.26) |
| **Diseases of the respiratory system** | 1.22 (1.13 to 1.32) |
| Influenza and pneumonia | 1.28 (1.14 to 1.44) |
| Chronic obstructive bronchitis | 1.72 (1.36 to 2.17) |
| Asthma | 1.37 (1.25 to 1.51) |

*eTable 10 continued from previous page*

| **Diseases of the digestive system** | 1.16 (1.09 to 1.22) |
| --- | --- |
| Appendicitis | 1.13 (0.96 to 1.34) |
| Inflammatory bowel disease | 1.22 (1.01 to 1.48) |
| Diseases of liver | 1.29 (0.98 to 1.70) |
| Alcoholic liver disease | 1.69 (1.13 to 2.51) |
| Pancreatitis | 1.26 (0.96 to 1.65) |
| **Diseases of the skin** | 1.17 (0.97 to 1.41) |
| Skin infections and eczema | 1.18 (0.91 to 1.54) |
| **Diseases of the musculoskeletal system** | 1.15 (1.02 to 1.29) |
| Rheumatoid arthritis and related disorders | 1.17 (1.12 to 1.23) |
| Gout | 1.05 (0.72 to 1.55) |
| Osteoarthritis | 1.13 (1.04 to 1.22) |
| Sciatica | 1.22 (1.05 to 1.43) |
| Back pain | 1.25 (0.99 to 1.57) |
| Soft tissue disorders | 1.19 (1.09 to 1.29) |
| **Diseases of the genitourinary system** | 1.13 (1.07 to 1.20) |
| Renal failure | 1.17 (0.85 to 1.61) |
| **Miscellaneous** |  |
| Circulatory and respiratory symptoms | 1.26 (1.09 to 1.45) |
| Digestive and abdominal symptoms | 1.38 (1.22 to 1.56) |
| Injury | 1.27 (1.19 to 1.35) |
| Poisoning | 2.83 (2.23 to 3.60) |
| Road accidents | 1.08 (0.87 to 1.34) |
| Falls | 1.32 (1.22 to 1.44) |

eFigure 4. Cumulative incidence of morbidity in individuals with versus without repeated physical or sexual abuse during childhood and adulthood (UK Biobank)


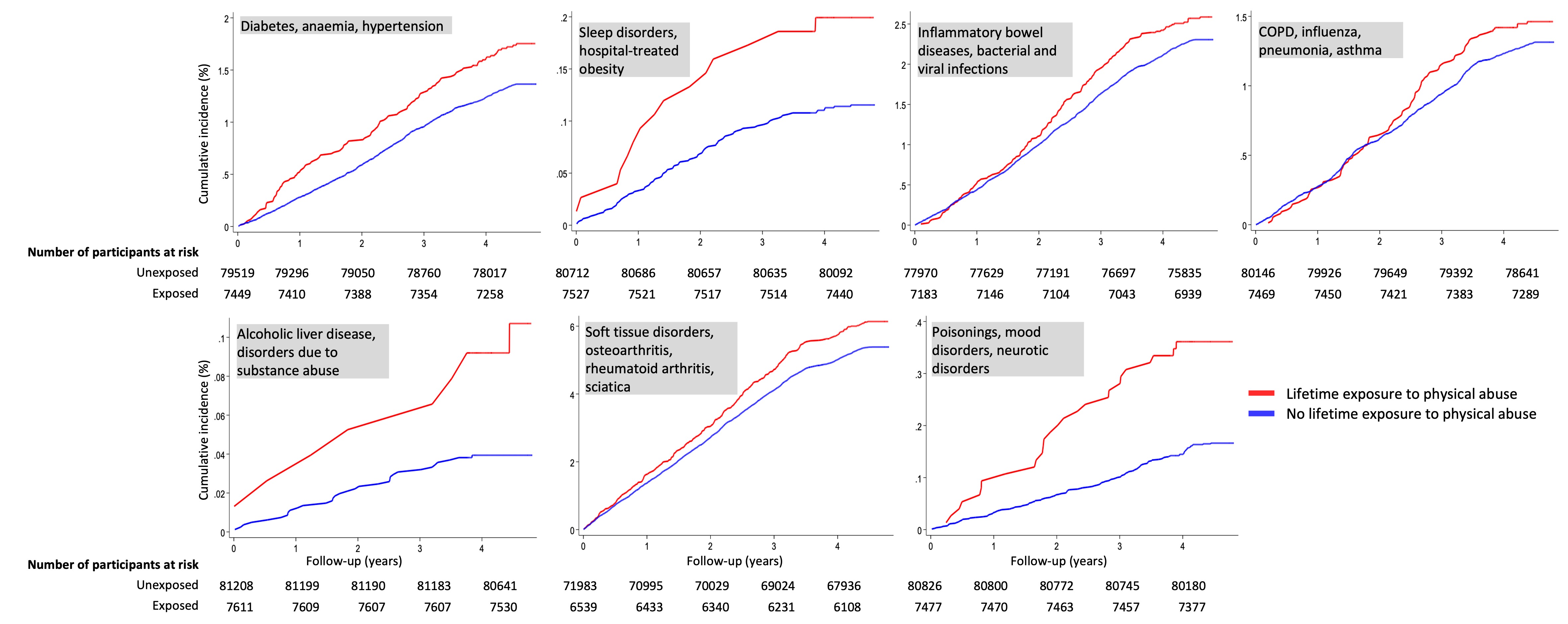


eTable 11. Statistical significance of the interaction term between childhood and adulthood abuse and logarithmically transformed follow-up time (UK Biobank)

eTable 12. Associations of abuse in childhood and adulthood with disease clusters before and after adjustment for the competing risk of death (Fine and Gray analysis in UK Biobank)

eTable 13. Depression as a mediator of the associations of childhood abuse, adulthood abuse, and repeated abuse across both life stages with mental and physical health outcomes after adjustment for age, sex, ethnic/racial origin, education, physical neglect, and emotional neglect

*Note:* Data on childhood physical and emotional neglect were ascertained from two questions taken from the brief Childhood Trauma Questionnaire. These enquiries ascertained if individuals had felt loved (“I felt love”) and/or taken to the doctor if needed (“There was someone to take me to the doctor if I needed it”). We computed separate binary variables (yes/no) for each abuse item such that a score of 1 or higher indicated that the person had experienced abuse, while a score of zero represented the reference group of individuals who had not been exposed to such adversities. The original response scale is 0 “never true”, 1 “rarely true”, 2 “sometimes true”, and 4 “very of true”. Emotional neglect: yes = < 2; physical neglect: yes = < 3.

eTable 14. Inflammation as a mediator of the associations of childhood abuse, adulthood abuse, and repeated abuse across both life stages with mental and physical health outcomes after adjustment for age, sex, ethnic/racial origin, education, and obesity

eTable 15. Age- and sex-adjusted associations of physical and emotional neglect during childhood with mental and physical health conditions in UK Biobank

|  | | **Hazard ratio (95% CI)** | | | |
| --- | --- | --- | --- | --- | --- |
| **Disease outcome** | | **Childhood physical neglect** | | **Childhood emotional neglect** | |
| **Infections** | | 1.35 (1.09 to 1.67) | | 1.34 (1.16 to 1.53) | |
| Bacterial infections | | 1.23 (0.96 to 1.57) | | 1.35 (1.16 to 1.57) | |
| Viral infections | | 1.62 (0.91 to 2.89) | | 1.37 (0.93 to 2.01) | |
| **Cancer** | | 1.07 (0.94 to 1.23) | | 1.07 (0.98 to 1.17) | |
| Colorectal cancer | | 0.75 (0.45 to 1.25) | | 1.01 (0.76 to 1.35) | |
| Lung cancer | | 1.32 (0.79 to 2.2) | | 2.12 (1.59 to 2.81) | |
| Melanoma | | 0.90 (0.71 to 1.15) | | 1.09 (0.94 to 1.26) | |
| Kidney cancer | | 1.01 (0.42 to 2.46) | | 1.25 (0.74 to 2.11) | |
| Brain cancer | |  | | 0.41 (0.13 to 1.29) | |
| Leukaemia, lymphoma | | 1.25 (0.79 to 1.97) | | 0.92 (0.66 to 1.3) | |
| **Diseases of the blood** | | 1.27 (0.99 to 1.63) | | 1.22 (1.03 to 1.43) | |
| Anaemia | | 1.20 (0.91 to 1.58) | | 1.25 (1.05 to 1.48) | |
| **Endocrine diseases** | | 1.54 (1.14 to 2.08) | | 1.13 (0.90 to 1.40) | |
| Diabetes | | 1.16 (0.43 to 3.12) | | 1.14 (0.60 to 2.17) | |
| Obesity requiring hospital treatment | |  | | 1.88 (0.80 to 4.41) | |
| **Mental and behavioural disorders** | | 2.89 (1.91 to 4.37) | | 1.98 (1.44 to 2.73) | |
| Dementia | | 3.19 (1.38 to 7.37) | | 1.68 (0.77 to 3.65) | |
| Disorders due to substance abuse | | 1.08 (0.15 to 7.83) | | 4.99 (2.53 to 9.86) | |
| Mood disorders | | 3.3 (1.33 to 8.18) | | 2.04 (1.02 to 4.1) | |
| Neurotic disorders | | 3.3 (1.53 to 7.12) | | 1.42 (0.72 to 2.82) | |
| Psychotic disorders | | 1.35 (0.18 to 9.91) | | 2.06 (0.72 to 5.85) | |
| **Diseases of the nervous system** | | 1.65 (1.34 to 2.03) | | 1.42 (1.23 to 1.63) | |
| Parkinson disease | | 1.96 (0.72 to 5.37) | | 0.66 (0.21 to 2.08) | |
| Multiple sclerosis | | 1.44 (0.2 to 10.49) | | 0.78 (0.19 to 3.26) | |
| Epilepsy | | 1.98 (0.62 to 6.28) | | 1.19 (0.48 to 2.95) | |
| Headaches | | 1.59 (0.82 to 3.09) | | 1.64 (1.11 to 2.45) | |
| TIA | | 1.2 (0.66 to 2.18) | | 1.03 (0.67 to 1.56) | |
| Sleep disorders | | 2.69 (1.42 to 5.1) | | 1.96 (1.23 to 3.12) | |
| **Diseases of the eye** | | 1.00 (0.87 to 1.15) | | 1.1 (1.01 to 1.21) | |
| **Diseases of the ear** | | 1.46 (0.91 to 2.33) | | 1.47 (1.09 to 1.98) | |
| **Diseases of the circulatory system** | | 1.29 (1.13 to 1.47) | | 1.15 (1.05 to 1.26) | |
| Hypertension | | 2.38 (1.44 to 3.95) | | 1.39 (0.91 to 2.11) | |
| Ischemic heart diseases | | 1.31 (1.06 to 1.63) | | 1.19 (1.02 to 1.38) | |
| Angina pectoris | | 1.33 (0.84 to 2.11) | | 1.47 (1.10 to 1.96) | |
| Myocardial infarction | | 0.84 (0.55 to 1.26) | | 1.33 (1.07 to 1.66) | |
| Pulmonary embolism | | 1.03 (0.61 to 1.76) | | 1.16 (0.84 to 1.59) | |
| Arrhythmias | | 1.14 (0.86 to 1.5) | | 1.18 (0.99 to 1.41) | |
| Heart failure | | 1.17 (0.68 to 2.04) | | 1.01 (0.67 to 1.52) | |
| Stroke | | 1.51 (1.1 to 2.09) | | 1.07 (0.83 to 1.37) | |
| Cerebrovascular diseases | | 1.56 (1.16 to 2.1) | | 1.09 (0.86 to 1.38) | |
| Intracerebral haemorrhage | | 1.56 (0.69 to 3.53) | | 1.01 (0.51 to 1.98) | |
| Cerebral infarction | | 1.47 (1.01 to 2.14) | | 1.03 (0.76 to 1.38) | |
| Arteriosclerosis | | 1.84 (0.68 to 5.03) | | 1.61 (0.78 to 3.32) | |
| Deep vein thrombosis | | 1.71 (1.07 to 2.75) | | 0.97 (0.66 to 1.43) | |
| **Diseases of the respiratory system** | | 1.37 (1.15 to 1.64) | | 1.50 (1.34 to 1.68) | |
| Influenza and pneumonia | | 1.37 (1.05 to 1.77) | | 1.32 (1.11 to 1.58) | |
| Chronic obstructive bronchitis | | 1.33 (0.76 to 2.3) | | 1.81 (1.30 to 2.51) | |
| Asthma | | 0.68 (0.22 to 2.12) | | 1.81 (1.15 to 2.85) | |

*eTable 15 continued from previous page*

| **Diseases of the digestive system** | 1.16 (1.05 to 1.28) | 1.27 (1.19 to 1.35) |
| --- | --- | --- |
| Appendicitis | 1.70 (0.9 to 3.19) | 1.64 (1.10 to 2.44) |
| Inflammatory bowel disease | 1.08 (0.73 to 1.59) | 1.44 (1.17 to 1.78) |
| Diseases of liver | 0.66 (0.25 to 1.78) | 1.19 (0.75 to 1.90) |
| Alcoholic liver disease | 1.64 (0.22 to 12.04) | 1.76 (0.53 to 5.81) |
| Pancreatitis | 2.30 (1.21 to 4.34) | 0.81 (0.43 to 1.54) |
| **Diseases of the skin** | 0.88 (0.70 to 1.11) | 0.99 (0.86 to 1.14) |
| Skin infections and eczema | 0.86 (0.56 to 1.33) | 1.05 (0.82 to 1.35) |
| **Diseases of the musculoskeletal system** | 1.37 (1.22 to 1.54) | 1.31 (1.22 to 1.41) |
| Rheumatoid arthritis and related disorders | 0.51 (0.23 to 1.14) | 1.31 (0.95 to 1.82) |
| Gout | 2.29 (0.71 to 7.32) | 1.57 (0.63 to 3.94) |
| Osteoarthritis | 1.18 (0.99 to 1.4) | 1.18 (1.06 to 1.32) |
| Sciatica | 1.86 (1.26 to 2.76) | 1.72 (1.33 to 2.22) |
| Back pain | 1.71 (1.24 to 2.35) | 1.86 (1.53 to 2.27) |
| Soft tissue disorders | 1.58 (1.3 to 1.93) | 1.39 (1.22 to 1.59) |
| **Diseases of the genitourinary system** | 1.19 (1.01 to 1.4) | 1.3 (1.18 to 1.43) |
| Renal failure | 1.16 (0.67 to 2.01) | 1.36 (0.97 to 1.91) |
| **Pregnancy complications** |  |  |
| Spontaneous abortion |  |  |
| Hypertension in pregnancy |  |  |
| Diabetes in pregnancy |  |  |
| Circulatory and respiratory symptoms | 1.31 (1.09 to 1.58) | 1.52 (1.36 to 1.69) |
| Digestive and abdominal symptoms | 1.29 (1.09 to 1.54) | 1.31 (1.18 to 1.46) |
| Injury | 1.41 (1.2 to 1.66) | 1.11 (0.99 to 1.25) |
| Poisoning | 2.04 (0.96 to 4.35) | 1.65 (0.98 to 2.76) |
| Road accidents |  |  |
| Falls | 2.51 (0.33 to 19.13) | 1.21 (0.16 to 9.20) |

eTable 16. Associations of physical and sexual abuse during childhood with mental and physical health conditions in UK Biobank – hazard ratios and 95% CIs adjusted for age, sex, emotional neglect, and physical neglect

|  | | **Hazard ratio (95% CI)** | | | |
| --- | --- | --- | --- | --- | --- |
| **Disease outcome** | | **Childhood physical abuse, additionally adjusted for physical and emotional neglect** | | **Childhood sexual abuse, additionally adjusted for physical and emotional neglect** | |
| **Infections** | | 1.14 (1.03 to 1.27) | | 1.23 (1.08 to 1.39) | |
| Bacterial infections | | 1.13 (1.01 to 1.27) | | 1.27 (1.11 to 1.45) | |
| Viral infections | | 1.08 (0.80 to 1.45) | | 1.02 (0.70 to 1.48) | |
| **Cancer** | | 0.99 (0.93 to 1.06) | | 1.04 (0.96 to 1.12) | |
| Colorectal cancer | | 1.05 (0.86 to 1.29) | | .91 (0.70 to 1.19) | |
| Lung cancer | | 1.56 (1.22 to 1.98) | | 1.21 (0.89 to 1.65) | |
| Melanoma | | 0.90 (0.8 to 1.01) | | .99 (0.86 to 1.13) | |
| Kidney cancer | | 1.13 (0.76 to 1.69) | | 1.47 (0.94 to 2.30) | |
| Brain cancer | | 0.70 (0.37 to 1.31) | | 0.89 (0.43 to 1.83) | |
| Leukaemia, lymphoma | | 0.95 (0.74 to 1.21) | | 1.06 (0.80 to 1.41) | |
| **Diseases of the blood** | | 1.25 (1.11 to 1.4) | | 1.41 (1.23 to 1.62) | |
| Anaemia | | 1.32 (1.16 to 1.49) | | 1.48 (1.28 to 1.71) | |
| **Endocrine diseases** | | 1.27 (1.09 to 1.48) | | 1.30 (1.08 to 1.56) | |
| Diabetes | | 1.22 (0.77 to 1.93) | | 1.16 (0.65 to 2.06) | |
| Obesity requiring hospital treatment | | 1.12 (0.56 to 2.28) | | 2.60 (1.34 to 5.06) | |
| **Mental and behavioural disorders** | | 2.00 (1.56 to 2.55) | | 1.44 (1.06 to 1.96) | |
| Dementia | | 1.41 (0.75 to 2.68) | | 0.86 (0.34 to 2.15) | |
| Disorders due to substance abuse | | 2.86 (1.46 to 5.61) | | 1.77 (0.77 to 4.07) | |
| Mood disorders | | 3.35 (2.05 to 5.5) | | 2.01 (1.11 to 3.66) | |
| Neurotic disorders | | 2.02 (1.27 to 3.20) | | 1.35 (0.75 to 2.44) | |
| Psychotic disorders | | 1.18 (0.47 to 2.95) | | 1.28 (0.44 to 3.72) | |
| **Diseases of the nervous system** | | 1.20 (1.07 to 1.34) | | 1.16 (1.01 to 1.32) | |
| Parkinson disease | | 1.84 (1.01 to 3.35) | | 0.89 (0.36 to 2.22) | |
| Multiple sclerosis | | 1.04 (0.44 to 2.45) | | 0.52 (0.12 to 2.20) | |
| Epilepsy | | 1.14 (0.59 to 2.20) | | 0.85 (0.34 to 2.12) | |
| Headaches | | 1.30 (0.96 to 1.78) | | 1.66 (1.18 to 2.33) | |
| TIA | | 1.26 (0.94 to 1.68) | | 1.12 (0.78 to 1.61) | |
| Sleep disorders | | 1.45 (0.99 to 2.12) | | 1.39 (0.86 to 2.23) | |
| **Diseases of the eye** | | 1.02 (0.96 to 1.09) | | 1.05 (0.97 to 1.13) | |
| **Diseases of the ear** | | 1.45 (1.16 to 1.82) | | 1.16 (0.87 to 1.54) | |
| **Diseases of the circulatory system** | | 1.15 (1.08 to 1.23) | | 1.25 (1.16 to 1.35) | |
| Hypertension | | 1.43 (1.05 to 1.94) | | 1.6 (1.13 to 2.26) | |
| Ischemic heart diseases | | 1.10 (0.99 to 1.23) | | 1.22 (1.07 to 1.39) | |
| Angina pectoris | | 1.20 (0.95 to 1.51) | | 1.45 (1.12 to 1.87) | |
| Myocardial infarction | | 1.01 (0.85 to 1.21) | | 1.20 (0.98 to 1.48) | |
| Pulmonary embolism | | 1.04 (0.82 to 1.32) | | 1.31 (1.00 to 1.71) | |
| Arrhythmias | | 1.07 (0.93 to 1.22) | | 1.21 (1.03 to 1.42) | |
| Heart failure | | 1.26 (0.94 to 1.67) | | 1.16 (0.82 to 1.66) | |
| Stroke | | 1.13 (0.94 to 1.36) | | 1.13 (0.91 to 1.4) | |
| Cerebrovascular diseases | | 1.11 (0.94 to 1.32) | | 1.13 (0.92 to 1.39) | |
| Intracerebral haemorrhage | | 1.62 (1.05 to 2.51) | | 0.88 (0.46 to 1.69) | |
| Cerebral infarction | | 1.01 (0.81 to 1.26) | | 1.08 (0.83 to 1.40) | |
| Arteriosclerosis | | 0.87 (0.44 to 1.69) | | 1.04 (0.47 to 2.26) | |
| Deep vein thrombosis | | 1.28 (1.00 to 1.66) | | 1.19 (0.87 to 1.63) | |
| **Diseases of the respiratory system** | | 1.25 (1.15 to 1.37) | | 1.27 (1.14 to 1.41) | |
| Influenza and pneumonia | | 1.13 (0.98 to 1.30) | | 1.27 (1.09 to 1.49) | |
| Chronic obstructive bronchitis | | 1.67 (1.28 to 2.16) | | 1.36 (0.98 to 1.88) | |
| Asthma | | 1.19 (0.82 to 1.74) | | 1.12 (0.71 to 1.78) | |

*eTable 16 continued from previous page*

| **Diseases of the digestive system** | 1.11 (1.06 to 1.16) | 1.22 (1.15 to 1.29) |
| --- | --- | --- |
| Appendicitis | 1.16 (0.84 to 1.6) | 1.38 (0.96 to 2.00) |
| Inflammatory bowel disease | 1.11 (0.94 to 1.30) | 1.14 (0.93 to 1.39) |
| Diseases of liver | 0.83 (0.57 to 1.21) | 0.95 (0.60 to 1.49) |
| Alcoholic liver disease | 1.04 (0.38 to 2.90) | 2.02 (0.69 to 5.9) |
| Pancreatitis | 1.07 (0.71 to 1.60) | 0.87 (0.51 to 1.48) |
| **Diseases of the skin** | 1.10 (1.00 to 1.21) | 1.10 (0.97 to 1.23) |
| Skin infections and eczema | 1.32 (1.12 to 1.56) | 1.12 (0.90 to 1.38) |
| **Diseases of the musculoskeletal system** | 1.17 (1.11 to 1.23) | 1.13 (1.05 to 1.2) |
| Rheumatoid arthritis and related disorders | 1.11 (0.86 to 1.42) | 0.95 (0.70 to 1.31) |
| Gout | 2.26 (1.19 to 4.29) | 1.09 (0.39 to 3.05) |
| Osteoarthritis | 1.13 (1.04 to 1.23) | 0.99 (0.89 to 1.10) |
| Sciatica | 1.22 (0.99 to 1.51) | 1.23 (0.96 to 1.59) |
| Back pain | 1.37 (1.17 to 1.61) | 1.49 (1.24 to 1.79) |
| Soft tissue disorders | 1.18 (1.07 to 1.31) | 1.21 (1.07 to 1.37) |
| **Diseases of the genitourinary system** | 1.18 (1.1 to 1.27) | 1.16 (1.06 to 1.26) |
| Renal failure | 1.34 (1.04 to 1.74) | 1.51 (1.12 to 2.03) |
| **Pregnancy complications** |  |  |
| Spontaneous abortion |  |  |
| Hypertension in pregnancy |  |  |
| Diabetes in pregnancy |  |  |
| Circulatory and respiratory symptoms | 1.30 (1.19 to 1.41) | 1.38 (1.25 to 1.52) |
| Digestive and abdominal symptoms | 1.23 (1.14 to 1.33) | 1.29 (1.17 to 1.41) |
| Injury | 1.17 (1.07 to 1.27) | 1.09 (0.99 to 1.20) |
| Poisoning | 1.73 (1.19 to 2.53) | 1.53 (0.97 to 2.42) |
| Road accidents |  |  |
| Falls | 2.06 (0.55 to 7.76) |  |

eTable 17. Associations of physical abuse during childhood with mental and physical health conditions in UK Biobank – hazard ratios and 95% CIs adjusted for age, sex, and sexual abuse

|  | | | **Hazard ratio (95% CI)** | |
| --- | --- | --- | --- | --- |
| **Disease outcome** | | **Childhood physical abuse** | |  |
| **Infections** | | 1.16 (1.05 to 1.27) | |  |
| Bacterial infections | | 1.19 (1.08 to 1.32) | |  |
| Viral infections | | 0.97 (0.73 to 1.28) | |  |
| **Cancer** | | 1.10 (1.04 to 1.16) | |  |
| Colorectal cancer | | 0.97 (0.80 to 1.16) | |  |
| Lung cancer | | 1.70 (1.38 to 2.10) | |  |
| Melanoma | | 1.07 (0.97 to 1.18) | |  |
| Kidney cancer | | 1.51 (1.10 to 2.07) | |  |
| Brain cancer | | 1.13 (0.71 to 1.80) | |  |
| Leukaemia, lymphoma | | 1.09 (0.89 to 1.33) | |  |
| **Diseases of the blood** | | 1.20 (1.08 to 1.33) | |  |
| Anaemia | | 1.23 (1.10 to 1.38) | |  |
| **Endocrine diseases** | | 1.21 (1.05 to 1.38) | |  |
| Diabetes | | 0.90 (0.59 to 1.38) | |  |
| Obesity requiring hospital treatment | | 2.12 (1.17 to 3.82) | |  |
| **Mental and behavioural disorders** | | 1.23 (0.96 to 1.57) | |  |
| Dementia | | 1.21 (0.67 to 2.18) | |  |
| Disorders due to substance abuse | | 1.39 (0.72 to 2.68) | |  |
| Mood disorders | | 1.09 (0.62 to 1.92) | |  |
| Neurotic disorders | | 1.55 (1.00 to 2.42) | |  |
| Psychotic disorders | | 0.98 (0.39 to 2.42) | |  |
| **Diseases of the nervous system** | | 1.22 (1.10 to 1.34) | |  |
| Parkinson disease | | 1.43 (0.83 to 2.46) | |  |
| Multiple sclerosis | | 0.98 (0.42 to 2.25) | |  |
| Epilepsy | | 0.62 (0.31 to 1.26) | |  |
| Headaches | | 1.44 (1.08 to 1.91) | |  |
| TIA | | 1.02 (0.78 to 1.33) | |  |
| Sleep disorders | | 1.12 (0.78 to 1.60) | |  |
| **Diseases of the eye** | | 1.05 (0.99 to 1.11) | |  |
| **Diseases of the ear** | | 1.18 (0.95 to 1.46) | |  |
| **Diseases of the circulatory system** | | 1.14 (1.08 to 1.21) | |  |
| Hypertension | | 1.17 (0.87 to 1.57) | |  |
| Ischemic heart diseases | | 1.13 (1.03 to 1.24) | |  |
| Angina pectoris | | 1.11 (0.90 to 1.37) | |  |
| Myocardial infarction | | 1.08 (0.93 to 1.25) | |  |
| Pulmonary embolism | | 1.03 (0.84 to 1.28) | |  |
| Arrhythmias | | 1.12 (1.00 to 1.26) | |  |
| Heart failure | | 0.91 (0.70 to 1.19) | |  |
| Stroke | | 1.07 (0.91 to 1.25) | |  |
| Cerebrovascular diseases | | 1.03 (0.89 to 1.20) | |  |
| Intracerebral haemorrhage | | 1.11 (0.73 to 1.69) | |  |
| Cerebral infarction | | 0.92 (0.76 to 1.12) | |  |
| Arteriosclerosis | | 0.84 (0.47 to 1.49) | |  |
| Deep vein thrombosis | | 0.98 (0.77 to 1.24) | |  |
| **Diseases of the respiratory system** | | 1.28 (1.18 to 1.38) | |  |
| Influenza and pneumonia | | 1.23 (1.09 to 1.38) | |  |
| Chronic obstructive bronchitis | | 1.41 (1.11 to 1.80) | |  |
| Asthma | | 1.41 (1.01 to 1.98) | |  |

*eTable 17 continued from previous page*

| **Diseases of the digestive system** | 1.15 (1.10 to 1.19) |
| --- | --- |
| Appendicitis | 1.05 (0.78 to 1.42) |
| Inflammatory bowel disease | 1.28 (1.10 to 1.48) |
| Diseases of liver | 1.08 (0.79 to 1.48) |
| Alcoholic liver disease | 1.23 (0.54 to 2.81) |
| Pancreatitis | 1.59 (1.14 to 2.20) |
| **Diseases of the skin** | 1.04 (0.95 to 1.13) |
| Skin infections and eczema | 1.03 (0.88 to 1.20) |
| **Diseases of the musculoskeletal system** | 1.24 (1.18 to 1.31) |
| Rheumatoid arthritis and related disorders | 1.18 (0.93 to 1.48) |
| Gout | 1.01 (0.52 to 1.96) |
| Osteoarthritis | 1.17 (1.08 to 1.26) |
| Sciatica | 1.22 (1.01 to 1.48) |
| Back pain | 1.58 (1.37 to 1.83) |
| Soft tissue disorders | 1.22 (1.12 to 1.34) |
| **Diseases of the genitourinary system** | 1.16 (1.08 to 1.23) |
| Renal failure | 1.16 (0.92 to 1.46) |
| **Pregnancy complications** | 3.56 (0.22 to 57.45) |
| Spontaneous abortion |  |
| Hypertension in pregnancy |  |
| Diabetes in pregnancy |  |
| Circulatory and respiratory symptoms | 1.14 (1.05 to 1.23) |
| Digestive and abdominal symptoms | 1.19 (1.11 to 1.28) |
| Injury | 1.09 (1.01 to 1.17) |
| Poisoning | 1.13 (0.77 to 1.66) |
| Road accidents |  |
| Falls | 0.84 (0.19 to 3.74) |

eTable 18. Frequencies and cases per abuse measure (childhood)

| **Disease outcome** | | | |  |  |  |  |  |  |  |  |
| --- | --- | --- | --- | --- | --- | --- | --- | --- | --- | --- | --- |
| **Disease category (ICD-10 chapter)** |  | **Diagnosis or diagnostic group*** | **ICD10-codes** | **Childhood physical abuse** | | **No childhood physical abuse** | | **Childhood sexual abuse** | | **No childhood sexual abuse** | |
|  |  |  |  | **N (cases)** | **N (cases)** | **N (cases)** | **N (total)** | **N (cases)** | **N (total)** | **N (cases)** | **N (total)** |
| **Infections** |  |  | **A01 - B89** | 594 | 29015 | 2237 | 124439 | 286 | 13254 | 2508 | 138753 |
|  |  | Bacterial infections | A01 - A79 | 513 | 29271 | 1881 | 125325 | 247 | 13377 | 2121 | 139756 |
|  |  | Viral infections | A80 - B34 | 67 | 29547 | 279 | 126371 | 32 | 13519 | 305 | 140923 |
| **Cancer** |  |  | **C00 - C97** | 1747 | 27255 | 7361 | 115029 | 749 | 12400 | 8283 | 128529 |
|  |  | Colorectal cancer | C18, C20 | 142 | 29568 | 689 | 126164 | 60 | 13545 | 762 | 140707 |
|  |  | Lung cancer | C34 | 123 | 29742 | 346 | 127026 | 47 | 13619 | 416 | 141658 |
|  |  | Melanoma | C43 - C44 | 555 | 28957 | 2473 | 123048 | 235 | 13278 | 2777 | 137286 |
|  |  | Breast cancer (women) | C50 | 225 | 14755 | 1023 | 70233 | 128 | 9268 | 1098 | 74569 |
|  |  | Prostate cancer (men) | C61 | 292 | 14028 | 1115 | 52011 | 86 | 3834 | 1318 | 61918 |
|  |  | Kidney cancer | C64 | 56 | 29736 | 157 | 126962 | 22 | 13618 | 187 | 141587 |
|  |  | Brain cancer | C71 | 23 | 29776 | 98 | 127153 | 8 | 13637 | 110 | 141799 |
|  |  | Leukaemia, lymphoma | C81 - C96 | 125 | 29615 | 522 | 126392 | 52 | 13555 | 591 | 140966 |
| **Diseases of the blood** |  |  | **D50 - D89** | 457 | 29033 | 1706 | 124156 | 245 | 13249 | 1892 | 138489 |
|  |  | Anaemia | D50 - D64 | 405 | 29187 | 1474 | 124811 | 223 | 13324 | 1634 | 139205 |
| **Endocrine diseases** |  |  | **E00 - E35** | 270 | 29219 | 1006 | 125236 | 135 | 13345 | 1128 | 139649 |
|  |  | Diabetes | E10 - E14 | 26 | 29711 | 124 | 126893 | 13 | 13612 | 136 | 141500 |
|  |  | Obesity requiring hospital treatment | E66 | 19 | 29729 | 33 | 127075 | 12 | 13610 | 38 | 141701 |
| **Mental and behavioural disorders** |  |  | **F00 - F99** | 85 | 29478 | 301 | 126419 | 48 | 13458 | 335 | 140971 |
|  |  | Dementia | F00 - F03, G30, G31 | 15 | 29777 | 63 | 127156 | 5 | 13641 | 71 | 141798 |
|  |  | Disorders due to substance abuse | F10 - F19 | 13 | 29721 | 33 | 127046 | 7 | 13610 | 39 | 141666 |
|  |  | Mood disorders | F30 - F39 | 17 | 29647 | 58 | 126892 | 14 | 13568 | 60 | 141492 |
|  |  | Neurotic disorders | F40 - F48 | 28 | 29702 | 78 | 126935 | 13 | 13577 | 93 | 141571 |
|  |  | Psychotic disorders | F20 - F29 | 6 | 29758 | 27 | 127104 | 4 | 13628 | 28 | 141746 |

*eTable 18 continued from previous page*

| **Disease outcome** |  |  |  |  |  |  |  |  |  |  |  |
| --- | --- | --- | --- | --- | --- | --- | --- | --- | --- | --- | --- |
| **Disease category (ICD-10 chapter)** |  | **Diagnosis or diagnostic group*** | **ICD10-codes** | **Childhood physical abuse** | | **No childhood physical abuse** | | **Childhood sexual abuse** | | **No childhood sexual abuse** | |
|  |  |  |  | **N (cases)** | **N (total)** | **N (cases)** | **N (total)** | **N (cases)** | **N (total)** | **N (cases)** | **N (total)** |
| **Diseases of the nervous system** |  |  | **G00 - G99** | 548 | 28049 | 2016 | 121087 | 249 | 12792 | 2270 | 134957 |
|  |  | Parkinson disease | G20 | 17 | 29775 | 62 | 127137 | 5 | 13639 | 74 | 141780 |
|  |  | Multiple sclerosis | G35 | 8 | 29739 | 30 | 127001 | 2 | 13620 | 34 | 141626 |
|  |  | Epilepsy | G40 - G42 | 9 | 29740 | 62 | 127001 | 5 | 13616 | 65 | 141634 |
|  |  | Headaches | G43 - G44 | 74 | 29642 | 196 | 126696 | 41 | 13569 | 222 | 141285 |
|  |  | TIA | G45 - G46 | 70 | 29679 | 314 | 126637 | 33 | 13578 | 347 | 141248 |
|  |  | Sleep disorders | G47 | 40 | 29454 | 143 | 126322 | 22 | 13495 | 160 | 140803 |
| **Diseases of the eye** |  |  | **H00 - H59** | 1425 | 27509 | 6916 | 116899 | 717 | 12496 | 7538 | 130540 |
| **Diseases of the ear** |  |  | **H60 - H99** | 118 | 29369 | 433 | 125498 | 55 | 13435 | 487 | 139964 |
| **Diseases of the circulatory system** |  |  | **I00 - I99** | 1553 | 25466 | 6312 | 110542 | 726 | 11741 | 7060 | 122989 |
|  |  | Hypertension | I10 - I15 | 60 | 29709 | 232 | 126882 | 39 | 13605 | 247 | 141496 |
|  |  | Ischemic heart diseases | I20 - I25 | 577 | 28389 | 2268 | 122049 | 250 | 13097 | 2570 | 135912 |
|  |  | Angina pectoris | I20 | 119 | 29297 | 485 | 125499 | 67 | 13430 | 531 | 139911 |
|  |  | Myocardial infarction | I21 | 229 | 29367 | 921 | 125525 | 100 | 13490 | 1041 | 139916 |
|  |  | Pulmonary embolism | I26 | 112 | 29613 | 482 | 126528 | 61 | 13561 | 527 | 141094 |
|  |  | Arrhythmias | I46 - I49 | 380 | 29124 | 1544 | 124350 | 173 | 13337 | 1724 | 138660 |
|  |  | Heart failure | I50 | 69 | 29720 | 355 | 126982 | 34 | 13622 | 385 | 141588 |
|  |  | Stroke | I60 - I61, I63 - I64 | 190 | 29560 | 867 | 126248 | 91 | 13529 | 959 | 140806 |
|  |  | Cerebrovascular diseases | I60 - I69 | 213 | 29504 | 1001 | 126036 | 104 | 13507 | 1102 | 140567 |
|  |  | Intracerebral haemorrhage | I61 | 27 | 29758 | 128 | 127074 | 10 | 13632 | 141 | 141706 |
|  |  | Cerebral infarction | I63 | 124 | 29656 | 653 | 126628 | 63 | 13578 | 711 | 141224 |
|  |  | Arteriosclerosis | I70 | 14 | 29739 | 78 | 127068 | 7 | 13631 | 84 | 141683 |
|  |  | Deep vein thrombosis | I80 - I82 | 85 | 29603 | 385 | 126441 | 45 | 13568 | 421 | 140987 |

*eTable 18 continued from previous page*

| **Disease outcome** |  |  |  |  |  |  |  |  |  |  |  |
| --- | --- | --- | --- | --- | --- | --- | --- | --- | --- | --- | --- |
| **Disease category (ICD-10 chapter)** |  | **Diagnosis or diagnostic group*** | **ICD10-codes** | **Childhood physical abuse** | | **No childhood physical abuse** | | **Childhood sexual abuse** | | **No childhood sexual abuse** | |
|  |  |  |  | **N (cases)** | **N (total)** | **N (cases)** | **N (total)** | **N (cases)** | **N (total)** | **N (cases)** | **N (total)** |
| **Diseases of the respiratory system** |  |  | **J00 - J99** | 860 | 27838 | 3082 | 120753 | 408 | 12833 | 3490 | 134370 |
|  |  | Influenza and pneumonia | J09 - J18 | 368 | 29429 | 1409 | 125817 | 178 | 13485 | 1580 | 140290 |
|  |  | Chronic obstructive bronchitis | J43 - J44, J47 | 92 | 29676 | 306 | 126853 | 44 | 13594 | 345 | 141449 |
|  |  | Asthma | J45 - J46 | 48 | 29598 | 150 | 126707 | 21 | 13560 | 175 | 141266 |
| **Diseases of the digestive system** |  |  | **K00 - K93** | 3195 | 20682 | 12559 | 91554 | 1542 | 9439 | 14064 | 101826 |
|  |  | Appendicitis | K35 | 58 | 29553 | 222 | 126279 | 34 | 13537 | 239 | 140819 |
|  |  | Inflammatory bowel disease | K50 - K52 | 250 | 28852 | 861 | 123857 | 113 | 13172 | 988 | 138104 |
|  |  | Diseases of liver | K70 - K77 | 53 | 29691 | 216 | 126808 | 21 | 13595 | 242 | 141415 |
|  |  | Alcoholic liver disease | K70 | 8 | 29773 | 22 | 127140 | 4 | 13635 | 26 | 141784 |
|  |  | Pancreatitis | K85 | 51 | 29672 | 149 | 126806 | 16 | 13587 | 180 | 141401 |
| **Diseases of the skin** |  |  | **L00 - L99** | 670 | 27573 | 2909 | 118417 | 322 | 12630 | 3224 | 131997 |
|  |  | Skin infections and eczema | L00 - L08, L20 - L30 | 207 | 29126 | 864 | 124889 | 99 | 13392 | 962 | 139172 |
| **Diseases of the musculoskeletal system** | |  | **M00 - M99** | 2139 | 23646 | 8284 | 104761 | 104761 | 991 | 10837 | 9306 |
|  |  | Rheumatoid arthritis and related disorders | M05-M06, M08, M13, M30-M35, M45 | 95 | 29543 | 405 | 126142 | 45 | 13504 | 445 | 140706 |
|  |  | Gout | M10 | 13 | 29759 | 44 | 127105 | 4 | 13636 | 51 | 141734 |
|  |  | Osteoarthritis | M15 - M19 | 920 | 27875 | 3857 | 119734 | 412 | 12787 | 4308 | 133435 |
|  |  | Sciatica | M50 - M51 | 148 | 29217 | 504 | 125495 | 73 | 13348 | 569 | 139887 |
|  |  | Back pain | M54 | 279 | 29059 | 765 | 124963 | 137 | 13285 | 891 | 139288 |
|  |  | Soft tissue disorders | M60 - M79 | 643 | 27626 | 2302 | 119416 | 297 | 12662 | 2600 | 132997 |

*eTable 18 continued from previous page*

| **Disease outcome** |  |  |  |  |  |  |  |  |  |  |  |
| --- | --- | --- | --- | --- | --- | --- | --- | --- | --- | --- | --- |
| **Disease category (ICD-10 chapter)** |  | **Diagnosis or diagnostic group*** | **ICD10-codes** | **Childhood physical abuse** | | **No childhood physical abuse** | | **Childhood sexual abuse** | | **No childhood sexual abuse** | |
|  |  |  |  | **N (cases)** | **N (total)** | **N (cases)** | **N (total)** | **N (cases)** | **N (total)** | **N (cases)** | **N (total)** |
| **Diseases of the genitourinary system** |  |  | **N00 - N99** | 1217 | 23915 | 4723 | 103909 | 552 | 10491 | 5321 | 116211 |
|  |  | Renal failure | N17 - N19 | 98 | 29701 | 365 | 126897 | 51 | 13608 | 405 | 141497 |
| **Pregnancy complications** |  |  | **O00 - O29** | 1 | 29151 | 1 | 125126 | 0 | 13327 | 2 | 139486 |
|  |  | Spontaneous abortion | O03 | 0 | 29642 | 1 | 126700 | 0 | 13574 | 1 | 141282 |
|  |  | Hypertension in pregnancy | O13 - O16 | 1 | 29711 | 0 | 126900 | 0 | 13607 | 1 | 141509 |
| **Miscellaneous** |  | Circulatory and respiratory symptoms | R00 - R09 | 832 | 27049 | 3221 | 117964 | 455 | 12322 | 3547 | 131343 |
|  |  | Digestive and abdominal symptoms | R10 - R19 | 1028 | 26837 | 3848 | 116368 | 541 | 12115 | 4267 | 129783 |
|  |  | Injury | S00 - T35 | 886 | 27184 | 3781 | 117541 | 437 | 12488 | 4196 | 130841 |
|  |  | Poisoning | T366 - T65 | 37 | 29519 | 129 | 126643 | 22 | 13477 | 141 | 141210 |
|  |  | Road accidents | V01 - V99 | 0 | 29783 | 2 | 127175 | 0 | 13641 | 2 | 141823 |
|  |  | Falls | W00 - W19 | 2 | 29783 | 13 | 127174 | 0 | 13641 | 15 | 141822 |

eTable 19. Frequencies and cases per abuse measure (adulthood)

| **Disease outcome** | | | |  |  |  |  |  |  |  |  |
| --- | --- | --- | --- | --- | --- | --- | --- | --- | --- | --- | --- |
| **Disease category (ICD-10 chapter)** |  | **Diagnosis or diagnostic group*** | **ICD10-codes** | **Adulthood physical abuse** | | **No adulthood physical abuse** | | **Adulthood sexual abuse** | | **No adulthood sexual abuse** | |
|  |  |  |  | **N (cases)** | **N (cases)** | **N (cases)** | **N (total)** | **N (cases)** | **N (total)** | **N (cases)** | **N (total)** |
| **Infections** |  |  | **A01 - B89** | 380 | 19523 | 2445 | 133800 | 186 | 8815 | 2639 | 144483 |
|  |  | Bacterial infections | A01 - A79 | 317 | 19709 | 2072 | 134756 | 152 | 8898 | 2237 | 145543 |
|  |  | Viral infections | A80 - B34 | 57 | 19904 | 288 | 135880 | 32 | 8987 | 312 | 146772 |
| **Cancer** |  |  | **C00 - C97** | 1077 | 18381 | 8031 | 123784 | 444 | 8309 | 8656 | 133839 |
|  |  | Colorectal cancer | C18, C20 | 106 | 19944 | 725 | 135660 | 54 | 9009 | 776 | 146574 |
|  |  | Lung cancer | C34 | 73 | 20038 | 396 | 136598 | 30 | 9059 | 439 | 147556 |
|  |  | Melanoma | C43 - C44 | 328 | 19560 | 2699 | 132315 | 115 | 8853 | 2908 | 143000 |
|  |  | Breast cancer (women) | C50 | 221 | 13994 | 1027 | 70879 | 114 | 8156 | 1130 | 76666 |
|  |  | Prostate cancer (men) | C61 | 89 | 5272 | 1318 | 60666 | 6 | 556 | 1402 | 65495 |
|  |  | Kidney cancer | C64 | 17 | 20042 | 196 | 136523 | 7 | 9054 | 206 | 147488 |
|  |  | Brain cancer | C71 | 14 | 20068 | 107 | 136728 | 7 | 9066 | 113 | 147706 |
|  |  | Leukaemia, lymphoma | C81 - C96 | 81 | 19956 | 569 | 135918 | 34 | 9022 | 614 | 146829 |
| **Diseases of the blood** |  |  | **D50 - D89** | 278 | 19554 | 1880 | 133502 | 134 | 8815 | 2022 | 144221 |
|  |  | Anaemia | D50 - D64 | 244 | 19671 | 1630 | 134193 | 118 | 8866 | 1757 | 144977 |
| **Endocrine diseases** |  |  | **E00 - E35** | 178 | 19699 | 1093 | 134627 | 93 | 8872 | 1177 | 145427 |
|  |  | Diabetes | E10 - E14 | 22 | 20027 | 129 | 136441 | 11 | 9041 | 140 | 147402 |
|  |  | Obesity requiring hospital treatment | E66 | 20 | 20039 | 31 | 136632 | 9 | 9054 | 42 | 147594 |
| **Mental and behavioural disorders** |  |  | **F00 - F99** | 61 | 19838 | 324 | 135939 | 44 | 8951 | 339 | 146795 |
|  |  | Dementia | F00 - F03, G30, G31 | 4 | 20071 | 74 | 136729 | 3 | 9067 | 75 | 147709 |
|  |  | Disorders due to substance abuse | F10 - F19 | 13 | 20021 | 33 | 136614 | 6 | 9051 | 40 | 147559 |
|  |  | Mood disorders | F30 - F39 | 18 | 19977 | 56 | 136436 | 12 | 9009 | 61 | 147375 |
|  |  | Neurotic disorders | F40 - F48 | 16 | 20004 | 90 | 136500 | 17 | 9034 | 89 | 147447 |
|  |  | Psychotic disorders | F20 - F29 | 7 | 20051 | 26 | 136682 | 4 | 9052 | 28 | 147656 |

*eTable 19 continued from previous page*

| **Disease outcome** |  |  |  |  |  |  |  |  |  |  |  |
| --- | --- | --- | --- | --- | --- | --- | --- | --- | --- | --- | --- |
| **Disease category (ICD-10 chapter)** |  | **Diagnosis or diagnostic group*** | **ICD10-codes** | **Adulthood physical abuse** | | **No adulthood physical abuse** | | **Adulthood sexual abuse** | | **No adulthood sexual abuse** | |
|  |  |  |  | **N (cases)** | **N (total)** | **N (cases)** | **N (total)** | **N (cases)** | **N (total)** | **N (cases)** | **N (total)** |
| **Diseases of the nervous system** |  |  | **G00 - G99** | 371 | 18855 | 2186 | 130162 | 174 | 8474 | 2384 | 140512 |
|  |  | Parkinson disease | G20 | 5 | 20072 | 71 | 136707 | 2 | 9066 | 76 | 147689 |
|  |  | Multiple sclerosis | G35 | 7 | 20044 | 31 | 136563 | 1 | 9048 | 37 | 147535 |
|  |  | Epilepsy | G40 - G42 | 11 | 20044 | 59 | 136565 | 8 | 9049 | 62 | 147536 |
|  |  | Headaches | G43 - G44 | 64 | 19945 | 206 | 136261 | 30 | 9001 | 240 | 147181 |
|  |  | TIA | G45 - G46 | 41 | 20004 | 345 | 136180 | 19 | 9044 | 366 | 147114 |
|  |  | Sleep disorders | G47 | 27 | 19884 | 156 | 135761 | 16 | 8990 | 168 | 146631 |
| **Diseases of the eye** |  |  | **H00 - H59** | 974 | 18509 | 7332 | 125771 | 453 | 8307 | 7853 | 135945 |
| **Diseases of the ear** |  |  | **H60 - H99** | 66 | 19783 | 485 | 134953 | 25 | 8937 | 527 | 145773 |
| **Diseases of the circulatory system** |  |  | **I00 - I99** | 878 | 17474 | 6975 | 118415 | 358 | 8010 | 7495 | 127849 |
|  |  | Hypertension | I10 - I15 | 38 | 20033 | 253 | 136425 | 20 | 9053 | 271 | 147381 |
|  |  | Ischemic heart diseases | I20 - I25 | 300 | 19409 | 2540 | 130895 | 105 | 8832 | 2737 | 141449 |
|  |  | Angina pectoris | I20 | 79 | 19824 | 526 | 134838 | 31 | 8972 | 575 | 145669 |
|  |  | Myocardial infarction | I21 | 114 | 19875 | 1033 | 134880 | 36 | 9017 | 1112 | 145715 |
|  |  | Pulmonary embolism | I26 | 61 | 19982 | 532 | 136026 | 32 | 9026 | 561 | 146957 |
|  |  | Arrhythmias | I46 - I49 | 215 | 19681 | 1706 | 133660 | 69 | 8928 | 1850 | 144388 |
|  |  | Heart failure | I50 | 38 | 20041 | 385 | 136528 | 18 | 9061 | 405 | 147484 |
|  |  | Stroke | I60 - I61, I63 - I64 | 124 | 19929 | 934 | 135746 | 45 | 9017 | 1014 | 146633 |
|  |  | Cerebrovascular diseases | I60 - I69 | 141 | 19893 | 1075 | 135514 | 55 | 9009 | 1162 | 146373 |
|  |  | Intracerebral haemorrhage | I61 | 14 | 20061 | 141 | 136637 | 3 | 9065 | 151 | 147609 |
|  |  | Cerebral infarction | I63 | 97 | 19992 | 681 | 136161 | 33 | 9038 | 747 | 147090 |
|  |  | Arteriosclerosis | I70 | 11 | 20054 | 80 | 136620 | 3 | 9060 | 88 | 147590 |
|  |  | Deep vein thrombosis | I80 - I82 | 45 | 19960 | 425 | 135951 | 25 | 9020 | 445 | 146868 |

*eTable 19 continued from previous page*

| **Disease outcome** |  |  |  |  |  |  |  |  |  |  |  |
| --- | --- | --- | --- | --- | --- | --- | --- | --- | --- | --- | --- |
| **Disease category (ICD-10 chapter)** |  | **Diagnosis or diagnostic group*** | **ICD10-codes** | **Adulthood physical abuse** | | **No adulthood physical abuse** | | **Adulthood sexual abuse** | | **No adulthood sexual abuse** | |
|  |  |  |  | **N (cases)** | **N (total)** | **N (cases)** | **N (total)** | **N (cases)** | **N (total)** | **N (cases)** | **N (total)** |
| **Diseases of the respiratory system** |  |  | **J00 - J99** | 562 | 18764 | 3377 | 129700 | 249 | 8508 | 3690 | 139935 |
|  |  | Influenza and pneumonia | J09 - J18 | 227 | 19816 | 1549 | 135296 | 116 | 8952 | 1659 | 146137 |
|  |  | Chronic obstructive bronchitis | J43 - J44, J47 | 76 | 19989 | 321 | 136408 | 35 | 9031 | 363 | 147345 |
|  |  | Asthma | J45 - J46 | 28 | 19942 | 170 | 136228 | 14 | 8991 | 184 | 147157 |
| **Diseases of the digestive system** |  |  | **K00 - K93** | 2140 | 13973 | 13591 | 98185 | 965 | 6361 | 14764 | 105776 |
|  |  | Appendicitis | K35 | 32 | 19923 | 246 | 135779 | 13 | 9000 | 266 | 146681 |
|  |  | Inflammatory bowel disease | K50 - K52 | 148 | 19418 | 961 | 133163 | 68 | 8735 | 1042 | 143821 |
|  |  | Diseases of liver | K70 - K77 | 42 | 20021 | 225 | 136346 | 22 | 9040 | 246 | 147303 |
|  |  | Alcoholic liver disease | K70 | 5 | 20068 | 25 | 136712 | 3 | 9067 | 27 | 147689 |
|  |  | Pancreatitis | K85 | 28 | 20004 | 172 | 136338 | 9 | 9031 | 190 | 147286 |
| **Diseases of the skin** |  |  | **L00 - L99** | 471 | 18562 | 3105 | 127299 | 200 | 8386 | 3379 | 137455 |
|  |  | Skin infections and eczema | L00 - L08, L20 - L30 | 157 | 19633 | 913 | 134249 | 63 | 8886 | 1007 | 144970 |
| **Diseases of the musculoskeletal system** | |  | **M00 - M99** |  | 1436 | 15905 | 8978 | 112404 | 641 | 7175 | 9776 |
|  |  | Rheumatoid arthritis and related disorders | M05-M06, M08, M13, M30-M35, M45 | 82 | 19882 | 417 | 135673 | 43 | 8976 | 456 | 146558 |
|  |  | Gout | M10 | 6 | 20069 | 51 | 136662 | 1 | 9067 | 56 | 147640 |
|  |  | Osteoarthritis | M15 - M19 | 637 | 18869 | 4137 | 128627 | 306 | 8513 | 4470 | 138948 |
|  |  | Sciatica | M50 - M51 | 82 | 19675 | 567 | 134897 | 49 | 8893 | 602 | 145660 |
|  |  | Back pain | M54 | 175 | 19540 | 867 | 134350 | 76 | 8801 | 968 | 145069 |
|  |  | Soft tissue disorders | M60 - M79 | 400 | 18635 | 2539 | 128278 | 167 | 8434 | 2780 | 138462 |

*eTable 19 continued from previous page*

| **Disease outcome** |  |  |  |  |  |  |  |  |  |  |  |
| --- | --- | --- | --- | --- | --- | --- | --- | --- | --- | --- | --- |
| **Disease category (ICD-10 chapter)** |  | **Diagnosis or diagnostic group*** | **ICD10-codes** | **Adulthood physical abuse** | | **No adulthood physical abuse** | | **Adulthood sexual abuse** | | **No adulthood sexual abuse** | |
|  |  |  |  | **N (cases)** | **N (total)** | **N (cases)** | **N (total)** | **N (cases)** | **N (total)** | **N (cases)** | **N (total)** |
| **Diseases of the genitourinary system** |  |  | **N00 - N99** | 739 | 15391 | 5199 | 112334 | 320 | 6643 | 5617 | 121069 |
|  |  | Renal failure | N17 - N19 | 64 | 20030 | 400 | 136433 | 28 | 9054 | 435 | 147386 |
| **Pregnancy complications** |  |  | **O00 - O29** | 1 | 19472 | 1 | 134673 | 1 | 8712 | 1 | 145418 |
|  |  | Spontaneous abortion | O03 | 1 | 19940 | 0 | 136269 | 1 | 8993 | 0 | 147195 |
|  |  | Hypertension in pregnancy | O13 - O16 | 0 | 20014 | 1 | 136462 | 0 | 9031 | 1 | 147423 |
| **Miscellaneous** |  | Circulatory and respiratory symptoms | R00 - R09 | 593 | 18258 | 3457 | 126615 | 288 | 8256 | 3753 | 136599 |
|  |  | Digestive and abdominal symptoms | R10 - R19 | 732 | 17776 | 4137 | 125321 | 344 | 7897 | 4521 | 135176 |
|  |  | Injury | S00 - T35 | 622 | 18253 | 4041 | 126354 | 285 | 8288 | 4374 | 136295 |
|  |  | Poisoning | T366 - T65 | 32 | 19832 | 134 | 136201 | 19 | 8937 | 147 | 147070 |
|  |  | Road accidents | V01 - V99 | 0 | 20074 | 2 | 136751 | 0 | 9068 | 2 | 147733 |
|  |  | Falls | W00 - W19 | 2 | 20074 | 13 | 136750 | 1 | 9068 | 14 | 147732 |
